# Theoretical Studies on Structures, Properties and Dominant Debromination Pathways for Selected Polybrominated Diphenyl Ethers

**DOI:** 10.3390/ijms17060927

**Published:** 2016-06-16

**Authors:** Lingyun Li, Jiwei Hu, Xuedan Shi, Wenqian Ruan, Jin Luo, Xionghui Wei

**Affiliations:** 1 Guizhou Provincial Key Laboratory for Information System of Mountainous Areas and Protection of Ecological Environment, Guizhou Normal University, Guiyang 550001, China; lingyunli1989@126.com (L.L.); xuedanshi1991@163.com (X.S.); wenqianruan@yahoo.com (W.R.) luojin@gznu.edu.cn (J.L.); 2 Department of Applied Chemistry, College of Chemistry and Molecular Engineering, Peking University, Beijing 100871, China; xhwei@pku.edu.cn

**Keywords:** relativistic effects, debromination, pseudo-potentials, transition state, adiabatic electron affinity, vertical electron affinity

## Abstract

The B3LYP/6-311+G(d)-SDD method, which considers the relativistic effect of bromine, was adopted for the calculations of the selected polybrominated diphenyl ethers (PBDEs) in the present study, in which the B3LYP/6-311+G(d) method was also applied. The calculated values and experimental data for structural parameters of the selected PBDEs were compared to find the suitable theoretical methods for their structural optimization. The results show that the B3LYP/6-311+G(d) method can give the better results (with the root mean square errors (RMSEs) of 0.0268 for the C–Br bond and 0.0161 for the C–O bond) than the B3LYP/6-311+G(d)-SDD method. Then, the B3LYP/6-311+G(d) method was applied to predict the structures for the other selected PBDEs (both neutral and anionic species). The lowest unoccupied molecular orbital (LUMO) and the electron affinity are of a close relationship. The electron affinities (vertical electron affinity and adiabatic electron affinity) were discussed to study their electron capture abilities. To better estimate the conversion of configuration for PBDEs, the configuration transition states for BDE-5, BDE-22 and BDE-47 were calculated at the B3LYP/ 6-311+G(d) level in both gas phase and solution. The possible debromination pathway for BDE-22 were also studied, which have bromine substituents on two phenyl rings and the bromine on meta-position prefers to depart from the phenyl ring. The reaction profile of the electron-induced reductive debromination for BDE-22 were also shown in order to study its degradation mechanism.

## 1. Introduction

Polybrominated diphenyl ethers (PBDEs) are a class of persistent organic pollutants (POPs), which are used extensively as the additive flame retardant for electronic equipments, textiles, building materials, *etc.* [1,2,3]. POPs are the chemicals which are toxic, recalcitrant, bioaccumulative, and can undergo long-range atmospheric transport [1,2,3]. The primary toxic effects of PBDEs include the endocrine disruption, and the adverse influences on nervous, reproductive, and immune systems [4].

Over the last several decades, the use and misuse of PBDEs make them ubiquitous in the environment [1,2,3,4]. The added halogen atoms can reduce their water solubility and increase their lipid solubility. The lipophilicity can lead to their bioaccumulation, which can cause a significant danger to human health [5]. Thus, corresponding laws and regulations have been propounded to limit their use. In 2003, the Restriction of Hazardous Substances Directive (RoHS) was adopted by the European Union [6]. It is essential that the flame retardants (polybrominated biphenyls (PBB) and PBDEs) are displaced by safer alternatives.

The development of effective and feasible debromination methods are pivotal to the remediation for the contamination of PBDEs. There are mainly three methods which have been proposed for the degradation of PBDEs: microbial reduction [7], photochemical degradation [8] and chemical degradation [9,10]. *Sulfurospirillum multivorans* and *Dehalococcoides* species are the representative anaerobic bacteria for the microbial reduction of PBDEs [7]. For photodegradation, Fang *et al.* proposed that consecutive reductive debromination was the main pathway, and debromination firstly occurred on the more brominated phenyl ring [8]. Step-wise debromination pathway was the major degradation process for their chemical reduction [9,10]. In addition, the adsorption materials (such as graphene) can also be used to remove PBDEs [11].

The molecular structures and properties are highly useful for understanding the mechanism of debromination deeply. The physicochemical properties of PBDEs have a strong dependence on the bromine substitution pattern [12]. Although plenty of experimental studies for PBDEs have been performed [13,14,15,16,17,18,19], the availability of pure compounds and the experimental difficulties still limits the acquirement of a large quantity of useful data. The electrophilicity exhibit tremendous predictive potential, which be adequate in developing a complete theory of chemical reactivity [20]. With the development of computer science, theoretical calculations become available for most reaction systems. It is significantly important for environmental researchers to explore the dehalogenation behaviors of PBDEs via combining theoretical results and the experimental data.

Many researchers simulated structures and studied the properties of PBDEs using quantum chemical methods [21,22,23,24,25]. Most of their theoretical results are consistent with the experimental data. Compared to the traditional theoretical methods (e.g., coupled-cluster theory, configuration interaction theory and Møller-Plesset theory) with electron correlation, the methods based on density functional theory (DFT) includes the electron correlation with much less calculations [26]. The previous studies reported that the results of DFT models are better than those of semiempirical methods [27]. In comparison with the X-ray crystal structure, the observed order of precision was DFT > PM3 = AM1 > MNDO for 2,3,7,8-TCDD [25,28].

The relativistic effects will play a significant role in theoretical studies for the molecules containing heavy elements and transition metals, inner-shell electrons of which move at speeds approaching the speed of light. For heavy elements, small basis sets can even cause a large amount of calculations. Effective core potentials (ECPs) can reduce the number of the basis function by an approximate way for the calculation of electrons near the nucleus [29,30]. ECPs only treat valence electrons, therefore this treatment can deal with some relativistic effects, which includes two main types, pseudo potentials (PP) and model potentials (MP) [29,30].

Both LANL2DZ and SDD are the commonly used basis sets [31,32,33]. The SDD basis set combines DZ with the Stuttgart-Dresden ECP basis set, which can reduce the cost caused by the large number of electrons of the third row transition metals [34]. Sieffert and Bühl used SDD basis sets on Ru with the small-core Stuttgart-Dresden relativistic effective core potential [35]. “genecp”, as a Gaussian keyword, was used to define the mixed basis sets. Chan and Fournier reported that the results (metal-metal bond lengths, dissociation energies and harmonic frequencies) of the ECP calculations for Cu and Ag, BP86/SDD and B3P86/SDD, are in close agreement with experimental results [31].

Bromine, as a heavy element, can influence the properties of PBDEs by relativistic effects, which should be considered to improve the calculational accuracy. However, only a few studies on PBDEs considered the relativistic effect of bromine. Pan and Bian applied LANL2DZ basis set augmented with polarization functions and diffuse functions for Br to study the photodegradation reaction for nona-BDEs with the solvent effect (methanol) [36]. Pan *et al.* further adopted the above basis set for bromine to study the photodegradation reaction of BDE-209 in tetrahydrofuran (THF) [37]. In our previous studies, the pseudo-potential SDD basis set was used for Br atom and 6-31+G(d) basis set for C, H, O atoms to obtain molecular descriptors, which were used to predict the debromination rate constants for PBDEs in both gas-phase and solution [23,38].

Electron affinity (EA) is an important molecular property which plays vital roles in electron –transfer reaction [39]. When electrons attached to PBDEs, the dissociation of C–Br bonds occurred with the products of bromide anions and free radical fragments [40]. The lowest unoccupied molecular orbital (LUMO) and the electron affinity are of a close relationship [41]. The highly brominated PBDEs can be easily reduced due to the lower energies of LUMO [41]. The chlorinated aromatic hydrocarbons will obtain electrons easily with the sufficiently high EAs and their congeners can be identified by electron capture negative-ion chemical ionization mass spectrometry (ECNIC-MS) with the premise that the EAs should be greater than 0.5 eV [41,42,43]. Thus, the brominated aromatic hydrocarbons may be identified by ECNICI-MS, when the EAs are bigger than the threshold [42].

For 1,2,3,7,8-PCDD anions, the transition states were studied by Zhao *et al.* to investigate the regioselective dechlorination, which indicated that the more toxic reductive dechlorination products will be obtained for the relatively lower energy barrier [39]. Eloranta *et al.* reported that both rings for PBDEs are able to interconvert via their transition states [44]. The transition states are significant important to determine the interconversion of BDE congeners. Luo *et al.* studied the debromination pathway and transition state of BDE-21, which have the three bromine substituents on one phenyl ring [45].

Solvents have a significant influence on the properties for solute, thus the calculational results for PBDEs might show an obvious discrepancy between in gas phase and in solution. Polarizable continuum model (PCM) is based on the reaction field theory characterized by the dielectric constant ε [38]. The conductor-like polarizable continuum model (CPCM), as the development of the polarizable continuum model (PCM), has a much simpler formalism to deal with the complex systems [46]. The CPCM solves the electrostatic potential using the solvation charges instead of the electron density [38,46].

As is known, the dominant debromination pathway for PBDEs determined by experiments are expensive. Hu *et al.* used the optimized structures of anionic PBDEs to predict their dominant debromination pathway and the calculated results are consistent with the experimental data, which confirmed the reliability of the methods [22].

According to the previous reports, the PP method has not been carried out for bromine atom to study the structural parameters for PBDEs [29,30,31]. In the present study, the structural parameters calculated for the selected PBDEs were compared with experimental data [14,15,16,17,18,19]. Both the B3LYP/6-311+G(d) method and B3LYP/genecp (6-311+G(d) for C, H, O atoms and SDD for Br atom) method were adopted for the calculations. The B3LYP functional (Becke exchange functional, three-parameter; Lee-Yang-Parr correlation functional) is one of the most widely used hybrid DFT methods [47,48,49]. The split valence 6-311+G(d) basis set, including the addition of diffuse functions, can deal with all electrons without considering the relativistic effect of Br [45]. Thus, the mixed basis set (genecp (6-311+G(d) for C, H, O atoms and SDD for Br atom)) was used to include the relativistic effect by use of the effective core potential for the inner electrons of Br. The equilibrium structures for the selected PBDEs (both neutral and anionic species) were calculated using the B3LYP/6-311+G(d) method. The C–H, C–O, C–Br bond lengths and the bond angles for the different PBDE congeners were compared. The dihedral angles of C–Br bonds for selected anionic PBDEs at the B3LYP/6-311+G(d) level were studied. The calculated electron affinities (vertical electron affinity and adiabatic electron affinity) for the selected PBDEs were discussed to study their helectron capture abilities. The transition states for BDE-5, BDE-22, and BDE-47 were demonstrated in both gas phase and solution, and the relationships between the total energy and the C–Br bond lengths for their anionic species were analyzed using the potential energy surfaces (PES), as shown in Figures S1–S3 (Supplementary Material). Since the bromine substituents for BDE-22 are on the different phenyl rings, their structural conversion may occur together with the electronic transfer across the two phenyl rings. The possible debromination pathway and the reaction profile of the electron-induced reductive debromination for BDE-22 were also shown in order to study its degradation mechanism. The PBDE congeners investigated in this study are listed in Table 1.

## 2. Results and Discussion

### 2.1. Molecular Geometry of Selected PBDEs

#### 2.1.1. Comparison of Calculated Structural Parameters with Experimental Values for PBDEs

**Figure 1:**
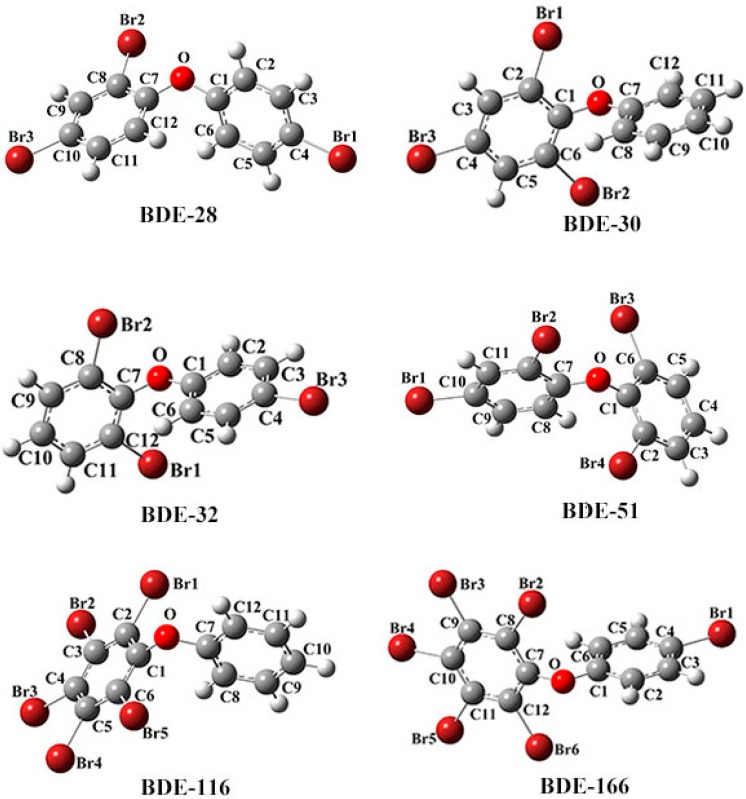
The molecular structures for BDE-28, BDE-30, BDE-32, BDE-51, BDE-116 and BDE-166, with the atom-numbering scheme.

The structural parameters for BDE-28, BDE-30, BDE-32, BDE-51, BDE-116, and BDE-166 calculated at the B3LYP/6-311+G(d) and B3LYP/genecp (6-311+G(d) for C, H, O atoms and SDD for Br) levels are shown in Table 2 and Table 3, and their atom-numbering schemes are shown in Figure 1. We also listed their observed structural parameters in Table 2 and Table 3, which were obtained from the references [14,15,16,17,18,19]. A good agreement can be found between the calculated values and observed data with the root mean square errors (RMSEs) of 0.0268 and 0.0161 for bond lengths of C–Br bond and C–O bond at the B3LYP/6-311+G(d) level, respectively. The RMSEs are 0.0511 and 0.0189 for bond lengths of C–Br bond and C–O bond at the B3LYP/genecp (6-311+G(d) for C, H, O atoms and SDD for Br atoms) level, respectively. As shown in Table 2 and Table 3, most of the values calculated at the two levels are bigger than the observed values for C–Br bond. However, all of the calculated values are smaller than the observed values for the C–O bond. Comparison of the results exhibits that the values of C–O bonds are more accurate than those of C–Br bonds calculated at the all-electron basis set (6-311+G(d)) and valent electron basis set (SDD) levels. The present study shows that the all-electron basis set 6-311+G(d) has advantages for the calculation of bond length of PBDEs. The SDD effective core potential basis set is not suitable for the bond length calculation for PBDEs. The results of the two methods are close to the experimental data, while the all-electron basis set is more suitable for the bond length calculation for PBDEs than the SDD effective core potential basis set. The results show that the calculated C–O bond lengths are more accurate than calculated C–Br bond lengths for PBDEs, which might be caused by the relativistic effect of bromine. However, the ECP with SDD basis set cannot increase the calculational accuracy, thus the new methods which can deal with the relativistic effect for bromine are needed to be proposed.

#### 2.1.2. The Comparison of Structural Parameters between Neutral and Anionic Species of the Selected PBDEs

The structures of PBDEs are similar to those of thyroid hormones and female hormone, thus they can cause health problems [27]. It is obvious that the conformational properties are important to study their environmental behaviors. The conformational parameters can be obtained via X-ray crystallography. However, the pure compounds are difficult to obtain, since the experiments need strict conditions [12]. To make up the structural data of PBDE congeners, quantum chemical computation plays an important role.

**Figure 2:**
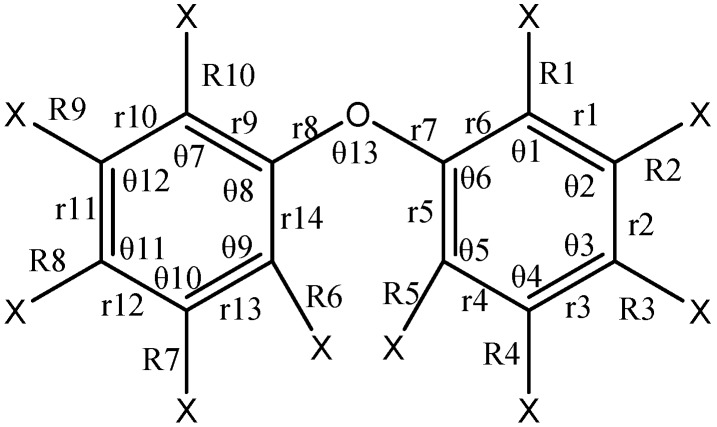
Atom numbering scheme for PBDEs (X = H or Br).

The earlier studies have confirmed that the structural parameters for PBDE congeners calculated at the B3LYP/6-31+G(d) level are reliable [22,42,45]. Average absolute errors between the calculated values and experimental values of bond length for the neutral persistent halogenated organic compounds (HOCs) are less than 0.04 Å [25]. Zhao *et al.* reported that the C–Br bond lengths from B3LYP/6-31+G(d) are 0.01 Å greater than the experimental values [42]. The present study reveals that the all-electron basis set (6-311+g(d)), considering the diffuse functions and the polarization function, is more suitable for the bond length calculation for PBDEs than the SDD effective core potential basis set. Thus, the structural parameters were investigated at the B3LYP/6-311+G(d) level in gas phase for the selected PBDEs, including 2,3,4′-tri-bromodiphenyl ether (BDE-22), 3,4′-di-bromodiphenyl ether (BDE-13), 2,4′-di-bromodiphenyl ether (BDE-8), 2,3-di-bromodiphenyl ether (BDE-5), 4-mono-bromodiphenyl ether (BDE-3), 3-mono-bromodiphenyl ether (BDE-2) and 2-mono-bromodiphenyl ether (BDE-1). The atom numbering scheme is shown in Figure 2.

The structural parameters of the selected neutral PBDE congeners and their corresponding anionic species are shown in Table 4 and Table 5 with C–Br bonds and angles in shadow. According to the calculated results, the coplanarity of diphenyl ether (DE) is practically impossible [42]. The C–Br bond length for the selected neutral PBDEs varies from 1.901 to 1.919 Å with slight differences. Except for BDE-13, the C–Br bond lengths for the selected PBDEs increase along with the distance of substitution position from the O atom, which is consistent with the previous theoretical studies [40,45]. The results show that the BDE congeners with more bromine substituents are mainly with shorter C–Br bond, which is in accordance with the earlier theoretical report [42].

The anionic species of PBDEs can be formed as a result of resonance capture of an electron by the molecules [21]. The added electron to the electron-rich phenyl ring clearly weakens the C–Br bond [42]. The length of elongated C–Br bond for anionic species with about 2.7 Å is approximate 0.7 Å longer than the other C–Br bonds. When the PBDE congeners capture an electron, the C–H bond and C–C bond also tend to elongate, as shown in Table 4 and Table 5. The elongated C–Br bonds of anionic species trend bending off the aromatic ring plane, as shown in Table 6.

The lengths of C–O bond vary from 1.368 to 1.389 Å for the selected neutral PBDEs, and vary from 1.361 to 1.411 Å for their anionic species. Comparing the bond lengths of the neutral PBDEs and their corresponding anionic species, we can find some rules. For the selected neutral PBDE congeners, it is interesting that the lengths of bond *r*8 are longer than bond *r*7, while the neutral BDE-3 congener has the opposite result. Thus, the bromine substituents probably have the effect on the shortening of the C–Br bond length.

For BDE-22, 13, 8, 5, and 1, the lengths of bond *r*7 of anionic structures are longer than those of neutral structures for BDE-22, 13, 8 and 5, while the lengths of bond *r*8 of anionic structures are shorter than those of neutral structures, except for BDE-3, which has the opposite result. The above data show that the electron attachment can also weaken the C–O bond.

### 2.2. Electron Affinities (EA) of the Selected PBDEs

When electrons attach to the halogenated compounds, the C–X bond tends to break and the compounds give bromide anions and free radical fragment [40]. Electron affinities (*EA*) include adiabatic electron affinities (*EA*_Ada_) and vertical electron affinities (*EA*_ver_), which can indicate the electron capture capacity [21]. Due to the rapid attachment of electron to the nucleus, anions are formed with the equilibrium geometry of the neutral molecule and the energies obtained are denoted as vertical attachment energies (*EA*_ver_) [40].

*EA*_Ada_ and *EA*_ver_ of the selected PBDEs were obtained using 6-311+G(d) with the B3LYP functional after zero-point energy (ZPE) correction, as shown in Table 7. To our knowledge, there are no published experimental EA values for BDE-22, 13, 8, 5, 3, 2, and 1. In the previous studies, DFT is always used to calculate the EA values for congers and the deviations between theoretical results and experimental data are in accord with the demands [21,42,43].

The values of *EA*_Ada_ calculated for the selected PBDEs were positive [42,43]. The electron capture negative-ion chemical ionization mass spectrometry (ECNICI-MS) can analyze the halogenated aromatic compounds [43]. According to the earlier studies, the threshold was calculated to be 0.5 eV for chlorinated aromatic compounds [50]. The values of *EA*_Ada_ in the present study are greater than 0.5 eV, thus the selected BDE congeners should be identified by ECNICI-MS [43].

As shown in Table 7, PBDEs with one bromine have negative values of the vertical electron affinity with ZPE correction, while those with two or three bromine have positive ones. These results are is agreement with the previous study [42]. For the *EA*_Ada_, geometry changes occur when the neutral species capture the electron, thus the *EA*_Ada_ values are significantly different from the *EA*_ver_ values [42]. It is obvious that the lengths of C–C bond and C–Br bond tend to be elongated, when the compounds capture an electron, as shown in Table 4 and Table 5, tending to yield molecular radical anions [42]. The increase in the number of bromine atoms (electron-withdrawing group) leads to the lowering of LUMO energy and hence the electron affinities of the PBDEs increase along with the increase in bromine number. This is in agreement with the previous theoretical studies [42,43].

The previous studies showed that *EA*_Ada_ may act as an indicator of toxicity for halogenated aromatic hydrocarbons [38,41]. PCDDs, PCBs, PCDFs, and PCDEs with the positive *EA* values act as electron acceptors in the reaction with receptors in living cells [38,41]. Thus, the PBDEs with the positive *EA*_Ada_ may also participate in electron-transfer reactions [42].

### 2.3. The Possible Anionic States and the Orbital Energies for the Selected BDE Congeners

**Figure 3:**
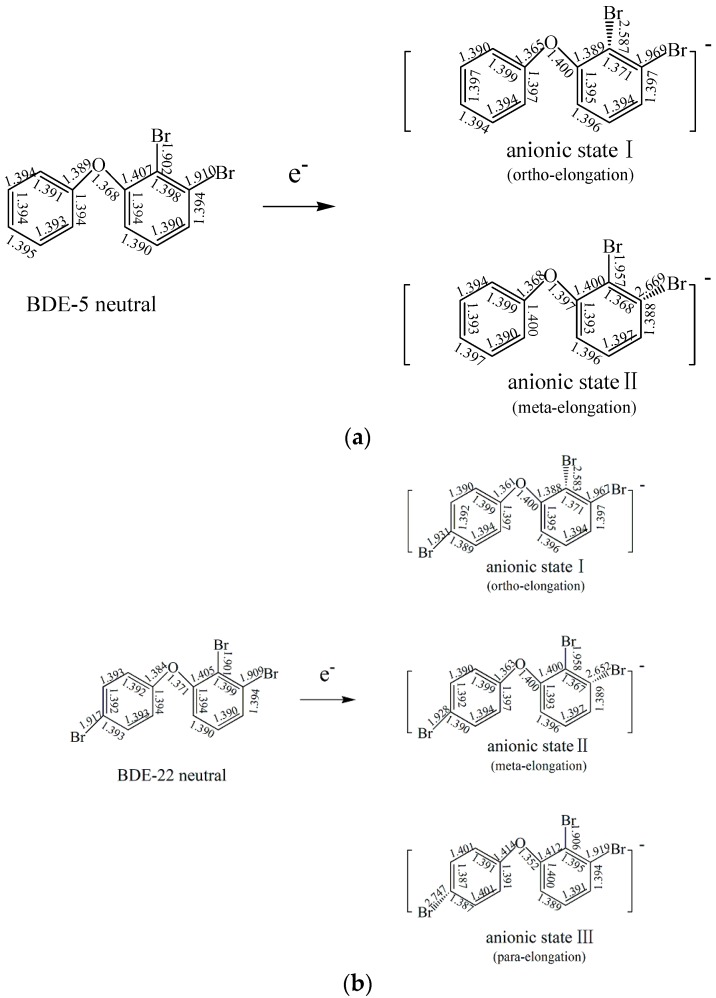

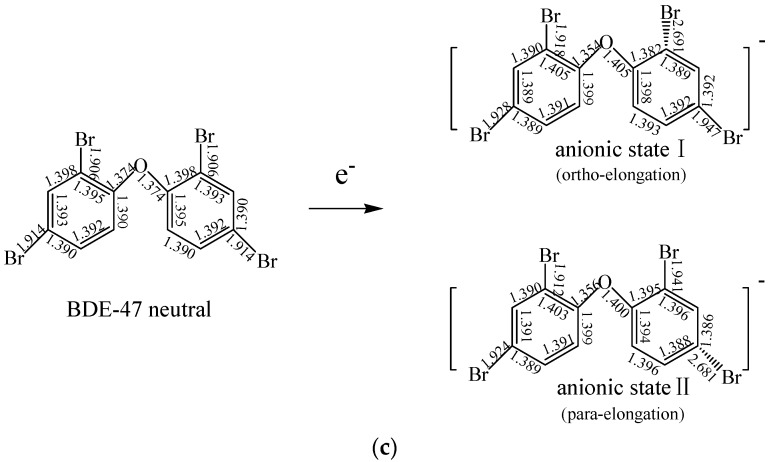
(**a**) Optimized geometries of BDE-5 and its possible anionic states; (**b**) Optimized geometries of BDE-22 and its possible anionic states; (**c**) Optimized geometries of BDE-47 and its possible anionic states.

The structural parameters of neutral BDE-5 and its possible anionic states obtained from the optimized geometries in this study are shown in Figure 3a. There are also big differences between the two BDE-5 anionic states (ortho- and meta-) and their neutral species. Both of the two C–Br bond of BDE-5 were elongated, when they capture the electrons. The C–Br bonds were elongated from *ca.* 1.901 Å for neutral species to *ca.* 2.652 Å for the anionic species.

**Figure 4:**
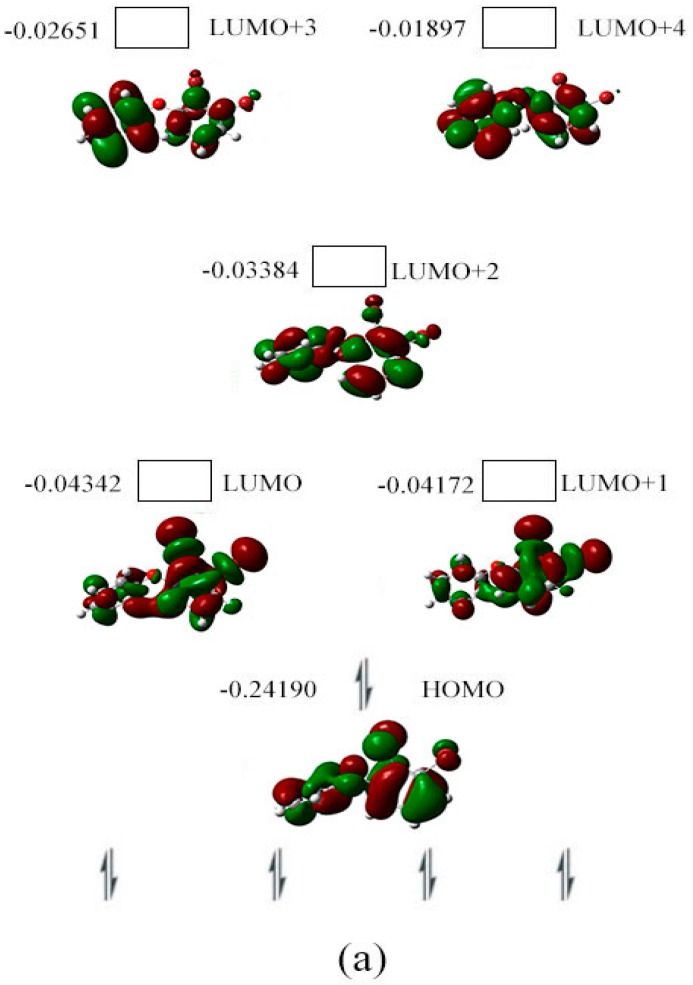

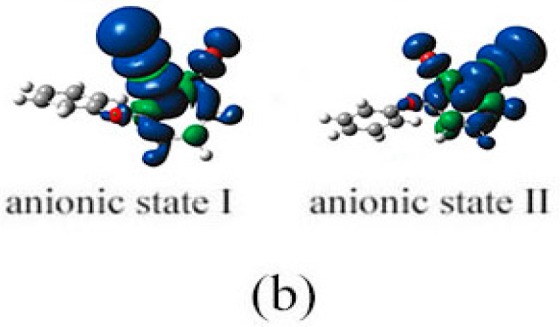
Orbital energy (hartree), HOMO, LUMO (**a**) iso-surface value = 0.02 au, degeneracy threshold = 0.01 hartree) for BDE-5 and its spin density (**b**) iso-surface value = 0.0004 au) surface image in gas phase.

As shown in Figure 4a, the HOMO (orbital 79) for the neutral species of BDE-5 is located on both phenyl groups. Its LUMO (orbital 80) is obviously located on the phenyl group with the substitution of two Br atoms. From orbital 79 to orbital 81 (HOMO to LUMO+1), the spatial distribution of orbital densities move from the two phenyl groups to the phenyl ring with the substitution of Br atoms. From orbital 82 to 84 (LUMO+2 to LUMO+4), the orbital densities is moved from the phenyl ring with the substitution of Br atom to the phenyl ring without the substitution of Br atoms. The spin densities of the specifical C–Br bond for the two possible anionic states are shown in Figure 4b. The Br atom captures an electron with the higher spin densities.

The addition of an electron weakens the C–Br bonds in PBDEs and the reductive debromination process may occur. The previous work studied the three possible anionic states of BDE-21 substituted by three Br atoms (ortho-, meta- and para- ) on the same phenyl ring [45]. To study the influence of bromine on the other phenyl ring, we selected BDE-22. Based on the stepwise debromination process, BDE-22 with the three Br substituents will have three possible anionic states (anionic state I, anionic state II and anionic state III).

**Figure 5:**
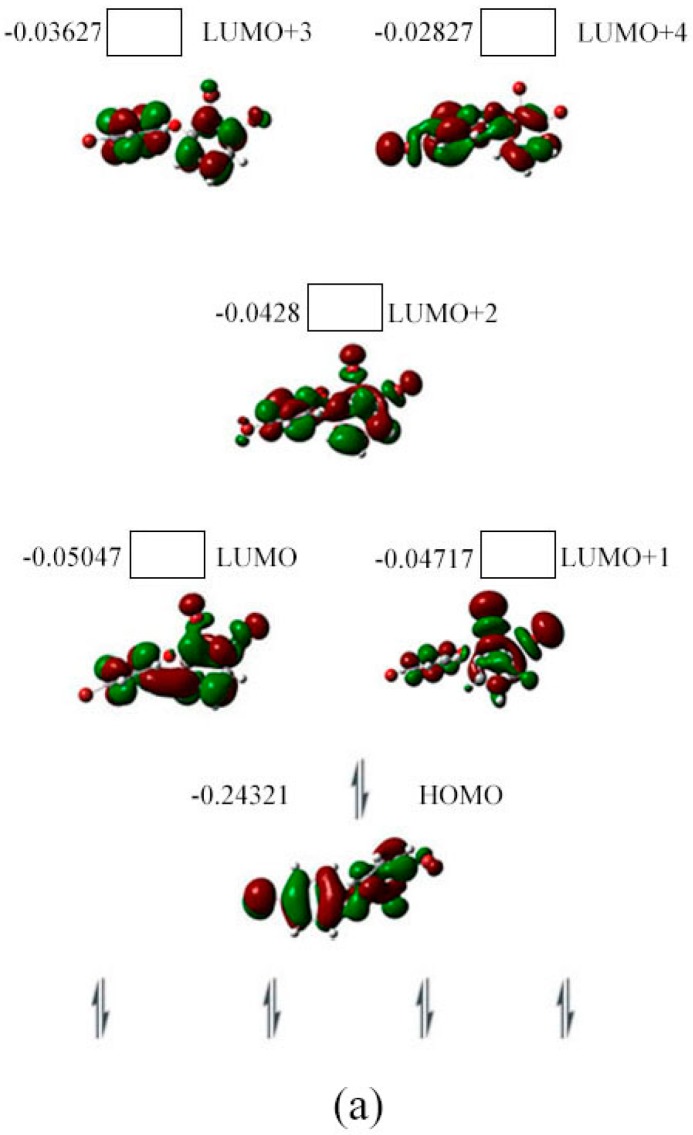

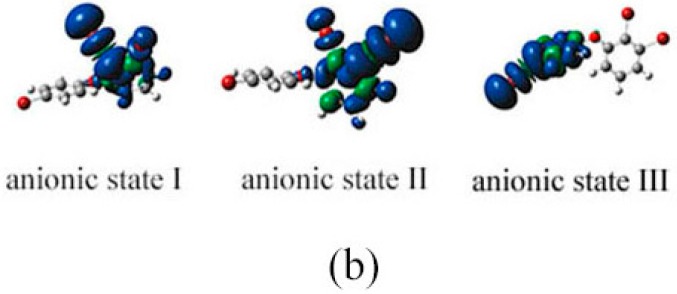
Orbital energy (hartree), HOMO, LUMO (**a**) iso-surface value = 0.02 au, degeneracy threshold = 0.01 hartree) for BDE-22 and its spin density (**b**) iso-surface value = 0.0004 au) surface image in gas phase.

The structural parameters of neutral BDE-22 and its possible anionic states obtained from the optimized geometries in the present study are shown in Figure 3b (Cartesian coordinates for the optimized structures from all of our calculations are shown in Figures S4–S6, Supplementary Material). There are significant differences between the three BDE-22 anionic states and their neutral species. All C–Br bond of the PBDE anions were somehow elongated by the additional electron in comparison to their corresponding neutral congeners. The C–Br bonds were elongated from *ca.* 1.91 Å for neutral BDE-22 to *ca.* 2.65 Å for the anionic states. The highest occupied molecular orbital (HOMO) for the neutral BDE-22 (orbital 96) is located on the two phenyl groups with all of the three C–Br bond. From orbital 96 to orbital 98 (HOMO to LUMO+1), the spatial distribution of orbital densities is gradually moved from the 4’-Br group to the 2-Br and 3-Br groups. From orbital 99 to orbital 101 (LUMO+2 to LUMO+4), the spatial distribution of orbital densities is oppositely moved from the 2-Br and 3-Br groups to the 4’-Br group, as shown in Figure 5a. The spin densities of the specifical C–Br bond for the three possible anionic states are shown in Figure 5b, which confirmed the electron attachment position.

The structural parameters of neutral BDE-47 and its two possible anionic states obtained from the optimized geometries in this study are shown in Figure 3c. Similar to BDE-22 and BDE-5, there are also significant differences between the two BDE-47 anionic states (ortho- and para-) and their neutral species, and the two C–Br bond of BDE-47 anionic states were elongated. The C–Br bonds were elongated from *ca.* 1.906 Å for neutral species to *ca.* 2.691 Å for the anionic species.

**Figure 6:**
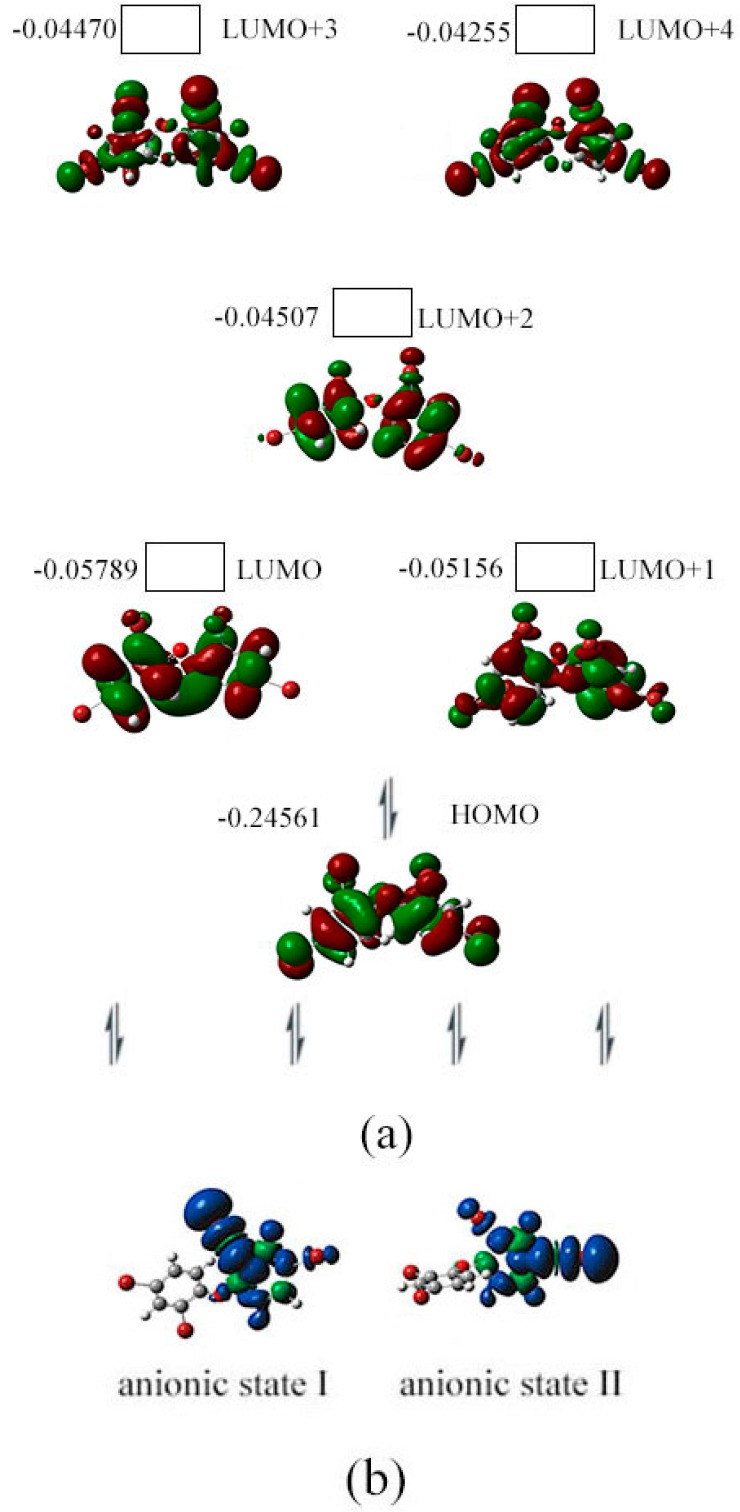
Orbital energy (hartree), HOMO, LUMO (**a**, iso-surface value = 0.02 au, degeneracy threshold = 0.01 hartree) for BDE-47 and its spin density (**b**, iso-surface value = 0.0004 au) surface image in gas phase.

The HOMO for the neutral species of BDE-47 (orbital 113) is located on the two phenyl groups with the π bond. Due to the symmetrical characteristic of BDE-47, the orbital densities is still located on the two phenyl rings from orbital 113 to 118 (HOMO to LUMO+4), as shown in Figure 6a. The spin densities of the specifical C–Br bond for the three possible anionic states are also shown in Figure 6a.

Compared with Table 7 and Table 8, it is obvious that the energies of lowest unoccupied molecular orbital (*E*_LUMO_) decrease along with the increase of the electron affinity. As shown in Table 8, the *E*_LUMO_ for PBDEs decrease along with an increase in the number of Br substituents. Thus, the electron capture process occurs more easily on the higher brominated PBDEs, which is consistent with the prior study [41].

PBDE congeners are conformationally flexible, thus the conversion of stable conformers can occur readily with low energy barriers [25]. The ideal way to identify the true energy minimum is to calculate the potential energy surface (PES), because the interconversion of the stable conformers for PBDEs can occur with low energy barriers [25,27]. The relationships between the total energy and the C–Br bond lengths of the anionic species for BDE-5, BDE-22, BDE-47 were analyzed using the PES, as shown in Figures S1–S3. The energy for the local minimum structure on the potential energy surface should be higher than that for the global minimum structure [21].

In the present study, the transition structure between possible anionic states for the selected PBDEs were calculated using QST2 method, which requires two molecule specifications, for the reactant and product, as its input [51,52]. The obtained Cartesian coordinates for the transition states from our calculations are found in the Appendix. They have an imaginary frequency with the stretching vibration of two elongated C–Br bonds.

**Figure 7:**
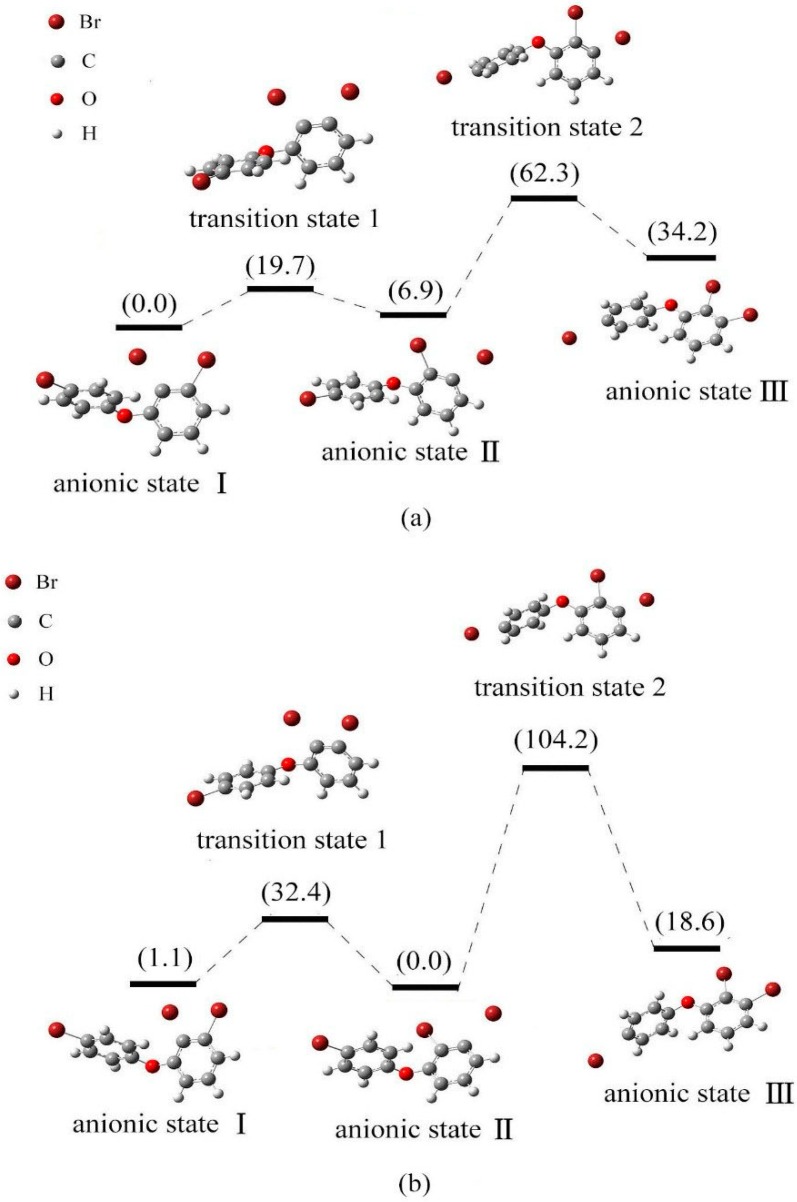
Relative energy of three possible anionic states and the transition states between them for BDE-22 (kJ/mol). ((**a**) in gas phase; (**b**) in solution).

In gas phase, the energy order of three anionic states was I < II < III, with the relative energy of 0.0, 6.9 and 34.2 kJ·mol^−1^, respectively. This conclusion is in agreement with the previous study, which is focused on BDE-21 [45]. Figure 7a shows the relative energy diagram of the anionic states I, II and III, and the transition states between them. The conversion of configuration for states I and II should overcome the energy barrier 19.7 kJ/mol, while conversion of states II to III need 62.3 kJ/mol energy is to overcome the energy barrier. Anionic states I and II are the relatively stable species with lower energy, which indicated that debromination of BDE-22 may occur at either the meta- or the ortho-position.

In solution, the energy order of three anionic states for BDE-22 was II < I < III, with the relative energy of 0.0, 1.1 and 18.6 kJ/mol, respectively. This conclusion is consistent with the previous study, which is focused on BDE-21 in solution [45]. Figure 7b shows the relative energy diagram of the anionic states I, II and III, and the transition states between them. The conversion of configuration for states I and II should overcome the energy barrier 32.4 kJ/mol, while isomerization of states II to III need 104.2 kJ/mol energy to overcome the conversion barrier. Anionic states I and II with low energy minima are the relatively stable species, and the debromination order of BDE-22 is meta-position > ortho-position > para-position in solution. This is consistent with the previous conclusion [45].

In addition, it is obvious that the energy barrier for states III and II are significantly larger than that for states I and II in both gas phase and solution for BDE-22. This may be attributable to the electronic transfer for one phenyl ring to the other ring for the conversion of states III and II. Thus, the structural conversion needs larger energy barrier for the electronic transfer across the two phenyl rings. Compared with the previous study [45], the results show that the energy barriers for the states I and II of BDE-22 and BDE-21 (in solution) are similar, while the energy barriers for the states III and II of BDE-22 and BDE-21 (in solution) are significantly different. This may also be ascribable to the electronic transfer across the phenyl rings.

**Figure 8:**
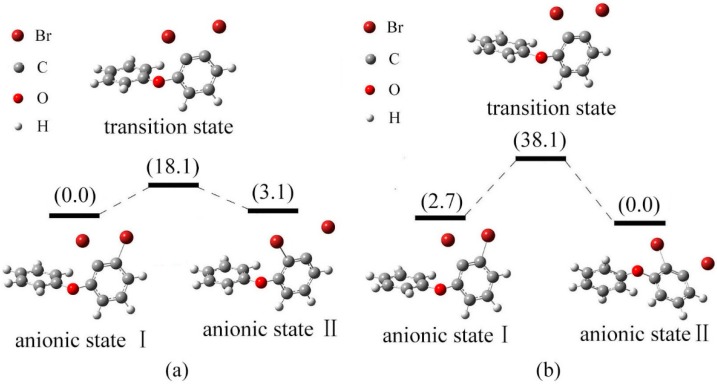
Relative energy of three possible anionic states and the transition states between them for BDE-5 (kJ/mol). ((**a**) in gas phase; (**b**) in solution).

Figure 8a shows the relative energies of the anionic states I, II for BDE-5 and their transition state in gas phase. The energy of anionic state II is higher than that of anionic state I by 3.1 kJ/mol. The conversion of configuration for states I and II should overcome the energy barrier 18.1 kJ/mol. Anionic state I is the relatively stable species. In solution, the energy order of two anionic states for BDE-5 was II < I, with the relative energy of 2.7 and 0.0 kJ/mol, respectively. As shown in Figure 8b, the conversion of configuration for states I and II should overcome the energy barrier 38.1 kJ/mol. Anionic state II with low energy minima is the relatively stable species, and the debromination order of BDE-5 is meta- position > ortho- position in solution. This is compatible with the previous conclusion [45].

**Figure 9:**
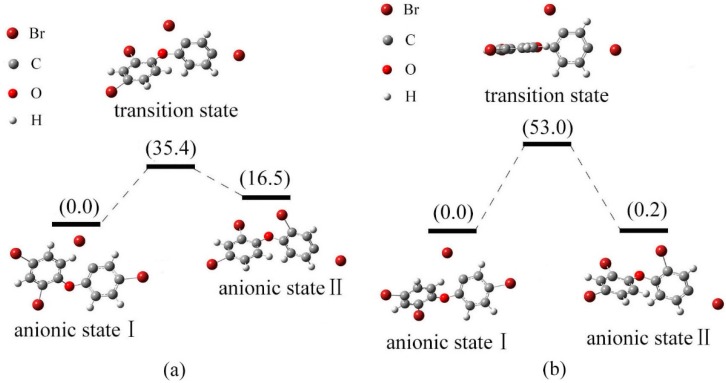
Relative energy of three possible anionic states and the transition states between them for BDE-47 (kJ/mol). ((**a**) in gas phase; (**b**) in solution).

The relative energies of the anionic states I, II for BDE-47 and their transition state in gas phase are shown in Figure 9a. The energy of anionic state II is higher than that of anionic state I by 6.5 kJ/mol. The conversion of states I to II needs 35.4 kJ/mol energy overcome the energy barrier. Anionic state I with low energy minima is the relatively stable, and this indicates that debromination of BDE-47 may occur at ortho-position. In solution, the energy order of two anionic states for BDE-47 was also I < II, with the relative energy of 0.0 and 0.2 kJ/mol, respectively. Figure 9b shows the relative energy diagram of the anionic states I, II and the transition state between them. The conversion of configuration for states I and II should overcome the energy barrier 53.0 kJ/mol. The bond cleavage of BDE-47 is at the meta- position both in gas phase and solution. This is compatible with the previous conclusion [45].

### 2.4. Use of Optimized Geometries of BDE Congeners to Predict the Dominant Debromimation Pathway

**Figure 10:**
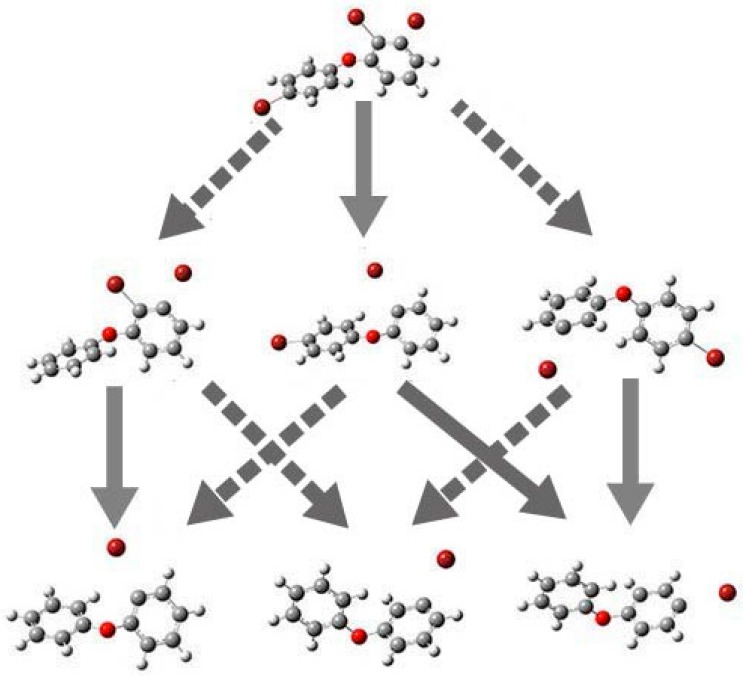
Geometries of the selected anionic BDE congeners optimized at the B3LYP/6-311+G(d) level and the proposed debromination pathway in gas phase (solid arrows indicated major pathways).

The earlier study has already confirmed that the B3LYP method is reliable to demonstrate on the debromination pathway [22]. The basis set 6-311+G(d) with diffusion function can reflect the anionic state properly. To the best of our knowledge, the debromination pathway for BDE-22 was not observed from the previous reports. The experimental data of debromination pathway for BDE-8 can be obtained from the study by Zhuang *et al.* [9]. When the neutral molecule capture an electron, the optimized geometry will change with one of the C–Br bond elongation. The elongated C–Br bond is probably the preferential cleavage bond via the electron inducted debromination mechanism. The theoretically calculated structural parameters of BDE-22, 13, 8, and 5 anionic states are listed in Table 5. Their dominant debromination pathway of BDE-22, 13, 8, and 5 is elucidated in Figure 10.

As shown in Figure 10, the results show that the cleavage of C–Br bond for BDE-22 occurred at the meta-position and this demonstrated that debromination prefers occurring at the meta-position. For BDE-5, the meta-bromine substituent also departs from the phenyl ring. For BDE-8, the ortho-position C–Br bond is more likely to break up than the para-position C–Br bond. These results show that the debromination position follows the orders: meta- > ortho- > para-, which is agreement with the previous studies. According to this rule, the amount of BDE-3 is larger than that of BDE-1 and BDE-2. In addition, the cleavage of the C–Br bond for PBDEs is more likely to occur in the ring with the higher bromination.

**Figure 11:**
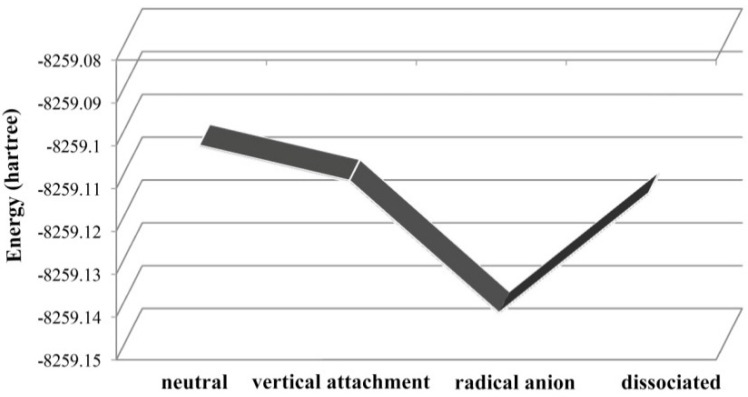
Reaction profile for electron-induced debromination of BDE-22 into the separated bromide anion and radical.

Interestingly, the C–O bond is so strong that there is no previous reports about the cleavage of this ether bond. The energy of each step for the debromination of BDE-22 is shown in Figure 11. When the neutral BDE-22 captures an electron, the stable anionic system with low energy minimum can be obtained (in gas-phase). Reaction profile for the electron-induced debromination of BDE-22 is shown in Figure 10. The anionic states with the optimized geometry has lower energy minimum than the vertical attachment energy, both of which capture an electron. Reaction profile for the electron-induced debromination of BDE-22 is shown in Figure 11. The radical anion with the optimized geometry has lower energy minimum than the vertical attachment energy, both of which capture an electron. The departing of bromine from the phenyl ring for BDE-22 needs 64.7 kJ/mol energy.

## 3. Materials and Methods

All calculations in the present study were performed with Gaussian 03 program suite [53], and GaussView 4.1 was used as the molecular modeling system to construct and visualize the results of the calculations. We chose DFT specified by the keyword B3LYP (Becke, three-parameter, Lee-Yang-Parr correlation functional) for all of the calculations [47,48]. We denoted the B3LYP/genecp (6-311+G(d) for C, H, O atoms and SDD for Br atom) method as the B3LYP/6-311+G(d)-SDD method. The molecular geometries of BDE-28, BDE-30, BDE-32, BDE-51, BDE-116 and BDE-166 were optimized at the B3LYP/6-311+G(d) level and the B3LYP/6-311+G(d)-SDD level.

Geometry optimizations were also performed at the B3LYP/6-311+G(d) level for BDE-1, BDE-2, BDE-3 BDE-5, BDE-13, and BDE-22. The transition states (TS) for BDE-22, BDE-5, and BDE-47 were also calculated at the B3LYP/6-311+G(d) level both in gas phase and solution. Methanol (ε = 32.613) was chosen as the solvent included in our calculations using the conductor-like polarizable continuum model (CPCM). All these optimized geometries were confirmed by frequency calculations to ensure that each of the geometries has the minimal energy. The 6-31+G(d) basis set was used to calculate the potential energy surface (PES) and intrinsic reaction coordinate (IRC) for BDE-22, BDE-5 and BDE-47. Their thermodynamic data (hartrees) calculated with B3LYP/6-311+G(d) in gas-phase. (Temperature of 298.150 Kelvin. Pressure of 1.00000 Atm) are shown in Tables S1–S3 (Supplementary Material).

The adiabatic electron affinities (*EA*_Ada_) and vertical electron affinities (*EA*_ver_) were obtained from the relationship as shown in Equations (1)–(2):

*EA*_Ada_ = *E*_neutral_ (optimized neutral) − *E*_anion_ (optimized anion) (1)

*EA*_Ver_ = *E*_neutral_ (optimized neutral) − *E*_anion_ (optimized neutral) (2)

Zero-point energies (ZPEs) for the correction of *EA*s were calculated with the harmonic vibrational frequencies.

## 4. Conclusions

The present study shows that the SDD effective core potential cannot effectively solve the relativistic effects of bromine for PBDEs. Thus, better methods are needed to simulate the relativistic effects of Br accurately. For the heavy elements (such as bromine), the description of PBDEs with the relativistic Dirac equation may obtain better results instead of the non-relativistic Schrödinger equation. The direct solution of the Dirac equation needs large calculations, which can alone deal with small molecules accurately. Zeroth-order regular approximation (ZORA) is a widely used approach for the relativistic calculation with small calculation and high precision, which is adopted to deal with the operator with simple formation [54]. ZORA Hamiltonian contains scalar-relativistic or spin-orbit coupling [55]. Pantazis and Neese calculated lanthanide series, actinide series with segmented all-electron relativistically contracted (SARC) basis sets, which are intended for use in combination with the popular Douglas-Kroll-Hess to the second order (DKH2) and ZORA scalar relativistic Hamiltonians. ZORA can perform well in the calculations of molecular properties [56,57]. A previous study on the Rh-Rh bond length for rhodium acetate exhibited that ZORA relativistic corrections could obtain the result consistent well with the experimental value [58]. The ZORA methods, DKH2 methods, and even the SARC basis set are needed to be used for PBDEs in the future studies, to deal with relativistic effects properly.

From the present theoretical study, it is obvious that the electron induced debromination for PBDEs have a significant role in the degradation of PBDEs, while the dominant pathway is the bromine departure. As is known, the lower brominated PBDEs have higher toxicity than PBDEs with higher bromination. The phenyl ring with π orbital is significantly solid, and the cleavage of ether bonds are also difficult. Thus, further in-depth theoretical studies need to be proposed to investigate the degradation of PBDEs.

## Figures and Tables

**Table 1:** IUPAC names and the congener numbers of the selected BDE congeners.

No.	IUPAC Name
BDE-166	2,3,4,4′,5,6-hexa-bromodiphenyl ether
BDE-116	2,3,4,5,6-penta-bromodiphenyl ether
BDE-51	2,2′,4,6′-tetra-bromodiphenyl ether
BDE-47	2,2′,4,4′-tetrabromodiphenyl ether
BDE-32	2,4′,6-tri-bromodiphenyl ether
BDE-30	2,4,6-tri-bromodiphenyl ether
BDE-28	2,4,4′-tri-bromodiphenyl ether
BDE-22	2,3,4′-tri-bromodiphenyl ether
BDE-13	3,4′-di-bromodiphenyl ether
BDE-8	2,4′-di-bromodiphenyl ether
BDE-5	2,3-di-bromodiphenyl ether
BDE-3	4-mono-bromodiphenyl ether
BDE-2	3-mono-bromodiphenyl ether
BDE-1	2-mono-bromodiphenyl ether

**Table 2:** Comparison of structural parameters for the selected PBDEs calculated at the B3LYP/6-311+G(d) and B3LYP/genecp (6-311+G(d)-SDD) levels with previous experimental values.

Congener	Bond Length	Exptl. (Å)	Reference ^a^	B3LYP/6-311+G(d)		Genecp	
				Calculated Value	Error	Calculated Value	Error
BDE-28	Br1–C4	1.901	[18]	1.917	0.016	1.945	0.044
	Br2–C8	1.889		1.907	0.018	1.933	0.044
	Br3–C10	1.889		1.915	0.026	1.942	0.053
BDE-30	Br1–C2	1.884	[14]	1.905	0.021	1.931	0.047
	Br2–C6	1.894		1.912	0.018	1.931	0.037
	Br3–C4	1.907		1.905	-0.002	1.939	0.032
BDE-32	Br1–C12	1.890	[15]	1.908	0.018	1.947	0.057
	Br2–C8	1.897		1.908	0.011	1.934	0.037
	Br3–C4	1.907		1.918	0.011	1.934	0.027
BDE-51	Br1–C10	1.895	[19]	1.915	0.020	1.943	0.048
	Br2–C12	1.902		1.906	0.004	1.932	0.030
	Br3–C6	1.885		1.907	0.022	1.933	0.048
	Br4–C2	1.879		1.907	0.028	1.933	0.054
BDE-116	Br1–C2	1.866	[17]	1.899	0.033	1.925	0.059
	Br2–C3	1.900		1.901	0.001	1.928	0.028
	Br3–C4	1.880		1.903	0.023	1.930	0.050
	Br4–C5	1.882		1.901	0.019	1.928	0.046
	Br5–C6	1.876		1.899	0.023	1.925	0.049
BDE-166	Br1–C4	1.844	[16]	1.917	0.073	1.945	0.101
	Br2–C8	1.878		1.899	0.021	1.925	0.047
	Br3–C9	1.890		1.900	0.010	1.927	0.037
	Br4–C10	1.865		1.902	0.037	1.929	0.064
	Br5–C11	1.883		1.900	0.017	1.927	0.044
	Br6–C12	1.845		1.899	0.054	1.925	0.080
RMSE				0.0268		0.0511	

^a^ The experimental data in the table were collected from the references [14,15,16,17,18,19].

**Table 3:** Comparison of structural parameters for the selected PBDEs calculated at the B3LYP/6-311+G(d) and B3LYP/genecp (6-311+G(d)-SDD) levels with previous experimental values.

Congener	Bond Length	Exptl. (Å)	Reference ^a^	B3LYP/6-311+G(d)		Genecp	
				Calculated Value	Error	Calculated Value	Error
BDE-28	O–C7	1.380	[18]	1.371	−0.009	1.370	−0.010
	O–C1	1.385		1.383	−0.002	1.383	−0.002
BDE-30	O–C1	1.381	[14]	1.365	−0.016	1.364	−0.017
	O–C7	1.394		1.391	−0.003	1.364	−0.030
BDE-32	O–C7	1.384	[15]	1.369	−0.015	1.368	−0.016
	O–C1	1.388		1.385	−0.003	1.385	−0.003
BDE-51	O–C7	1.395	[19]	1.375	−0.020	1.374	−0.021
	O–C1	1.397		1.371	−0.026	1.370	−0.027
BDE-116	O–C1	1.371	[17]	1.363	−0.008	1.362	−0.009
	O–C7	1.403		1.393	−0.010	1.393	−0.010
BDE-166	O–C7	1.391	[16]	1.365	−0.026	1.364	−0.027
	O–C1	1.414		1.389	−0.025	1.389	−0.025
RMSE				0.0161		0.0189	

^a^ The experimental data in the table were collected from the references [14,15,16,17,18,19].

**Table 4:** The structural parameters of the selected neutral PBDEs calculated at the B3LYP/6-311+G(d) level [*R* (Å), *r* (Å), and θ (deg)].

Bond Length	BDE-22	BDE-13	BDE-8	BDE-5	BDE-3	BDE-2	BDE-1
*R*1	1.901	1.082	1.910	1.902	1.084	1.082	1.911
*R*2	1.909	1.919	1.083	1.910	1.085	1.918	1.084
*R*3	1.083	1.083	1.084	1.082	1.085	1.083	1.084
*R*4	1.085	1.085	1.085	1.085	1.085	1.085	1.085
*R*5	1.083	1.084	1.084	1.083	1.084	1.083	1.084
*R*6	1.084	1.084	1.084	1.084	1.083	1.084	1.084
*R*7	1.083	1.083	1.083	1.085	1.083	1.085	1.085
*R*8	1.917	1.917	1.918	1.085	1.918	1.085	1.085
*R*9	1.083	1.083	1.083	1.085	1.083	1.085	1.085
*R*10	1.084	1.084	1.084	1.084	1.084	1.084	1.084
*r*1	1.399	1.391	1.393	1.398	1.394	1.389	1.392
*r*2	1.394	1.391	1.392	1.395	1.394	1.392	1.393
*r*3	1.390	1.395	1.394	1.390	1.395	1.394	1.394
*r*4	1.390	1.391	1.391	1.390	1.393	1.393	1.391
*r*5	1.394	1.394	1.397	1.394	1.395	1.395	1.397
*r*6	1.405	1.396	1.398	1.407	1.392	1.396	1.400
*r*7	1.371	1.379	1.374	1.368	1.386	1.376	1.371
*r*8	1.384	1.382	1.381	1.389	1.377	1.386	1.386
*r*9	1.392	1.392	1.393	1.391	1.395	1.392	1.392
*r*10	1.393	1.393	1.392	1.394	1.391	1.394	1.394
*r*11	1.392	1.392	1.392	1.394	1.392	1.394	1.394
*r*12	1.393	1.392	1.392	1.395	1.391	1.395	1.395
*r*13	1.393	1.393	1.393	1.393	1.394	1.393	1.393
*r*14	1.394	1.395	1.394	1.394	1.395	1.395	1.394
θ1	119.0	118.4	120.4	119.1	119.5	118.8	120.5
θ2	120.7	122.0	120.0	120.8	120.4	121.8	120.0
θ3	119.6	118.4	119.8	119.4	119.6	118.3	119.7
θ4	120.6	121.0	120.2	120.7	120.6	121.4	120.3
θ5	119.8	119.3	120.3	119.9	119.2	119.0	120.3
θ6	120.3	120.9	119.3	120.1	120.8	120.7	119.1
θ7	119.8	119.9	119.9	119.3	120.1	119.4	119.4
θ8	120.6	120.5	120.5	121.1	120.2	120.8	120.9
θ9	119.6	119.7	119.7	119.1	119.7	119.2	119.2
θ10	119.6	119.6	119.7	120.5	119.7	120.5	120.6
θ11	120.9	120.9	120.9	119.7	120.8	119.6	119.6
θ12	119.4	119.4	119.4	120.3	119.5	120.4	120.4
θ13	120.7	121.0	120.7	120.7	120.8	120.8	120.8

**Table 5:** The structural parameters of the selected anionic PBDEs calculated at the B3LYP/6-311+G(d) level [*R* (Å), *r* (Å), and θ (deg)].

Bond Length	BDE-22	BDE-13	BDE-8	BDE-5	BDE-3	BDE-2	BDE-1
*R*1	1.958	1.088	2.793	1.957	1.085	1.087	2.806
*R*2	2.635	2.754	1.086	2.668	1.086	2.786	1.086
*R*3	1.087	1.087	1.088	1.087	1.085	1.087	1.088
*R*4	1.088	1.089	1.087	1.088	1.086	1.089	1.087
*R*5	1.085	1.085	1.087	1.085	1.083	1.085	1.087
*R*6	1.083	1.083	1.085	1.083	1.088	1.085	1.085
*R*7	1.084	1.084	1.084	1.083	1.088	1.086	1.087
*R*8	1.928	1.928	1.933	1.085	2.780	1.085	1.086
*R*9	1.084	1.084	1.083	1.086	1.088	1.086	1.086
*R*10	1.084	1.085	1.081	1.085	1.088	1.083	1.081
*r*1	1.368	1.387	1.388	1.368	1.390	1.386	1.387
*r*2	1.389	1.388	1.399	1.388	1.397	1.387	1.399
*r*3	1.397	1.399	1.397	1.397	1.393	1.399	1.397
*r*4	1.396	1.398	1.393	1.396	1.395	1.398	1.393
*r*5	1.392	1.391	1.401	1.393	1.399	1.392	1.401
*r*6	1.400	1.395	1.382	1.400	1.401	1.396	1.383
*r*7	1.400	1.411	1.403	1.397	1.365	1.408	1.400
*r*8	1.364	1.361	1.365	1.368	1.409	1.365	1.369
*r*9	1.399	1.401	1.397	1.399	1.392	1.398	1.396
*r*10	1.390	1.390	1.395	1.390	1.402	1.395	1.394
*r*11	1.392	1.392	1.389	1.397	1.386	1.394	1.394
*r*12	1.389	1.389	1.391	1.393	1.386	1.397	1.396
*r*13	1.394	1.394	1.390	1.394	1.402	1.390	1.391
*r*14	1.397	1.399	1.401	1.397	1.392	1.401	1.401
θ1	120.4	118.5	119.6	120.5	120.1	118.5	119.7
θ2	120.4	121.2	120.5	120.5	120.6	121.4	120.5
θ3	119.9	119.6	119.7	119.7	119.0	119.5	119.7
θ4	119.9	120.3	119.8	119.9	121.1	120.3	119.8
θ5	119.6	118.7	119.7	119.7	119.5	118.8	119.8
θ6	119.8	121.8	120.7	119.6	119.7	121.6	120.5
θ7	120.5	120.6	119.6	120.0	119.0	119.4	119.1
θ8	119.7	119.5	119.7	119.9	121.3	119.8	119.9
θ9	119.9	119.9	120.7	119.4	119.0	120.1	120.3
θ10	119.8	119.9	118.9	121.1	119.9	120.6	120.3
θ11	120.9	120.8	121.0	119.0	120.7	119.0	119.0
θ12	119.3	119.3	120.0	120.6	119.9	121.2	121.4
θ13	119.6	119.7	123.3	119.8	119.4	119.9	123.1

**Table 6:** The dihedral angles of C–Br bond for the selected anionic PBDEs at the B3LYP/6-311+G(d) level (deg).

Congeners	Ortho-Position	Meta-Position	Para-Position
BDE-22	178.5	−165.7	179.9
BDE-13	-	−162.5	179.9
BDE-8	−174.1	-	179.5
BDE-5	179.4	−175.8	-
BDE-3	-	-	−173.2
BDE-2	-	166.5	-
BDE-1	−177.7	-	-

**Table 7:** Electron affinities (*EA*_Ada_ and *EA*_ver_) of the selected PBDEs.

*EA*		BDE-22	BDE-13	BDE-8	BDE-5	BDE-3	BDE-2	BDE-1
*EA*_Ada_	au	0.0392	0.0277	0.0306	0.0350	0.0230	0.0238	0.0250
	eV	1.066	0.753	0.832	0.951	0.626	0.647	0.681
*EA*_ver_	au	0.0082	0.0046	0.0029	0.0017	−0.0053	−0.0061	−0.0080
	eV	0.223	0.124	0.079	0.046	−0.145	−0.167	−0.218

**Table 8:** The orbital energies (hartree) of HOMO and LUMO for the selected neutral BDE congeners.

Orbit	BDE-47	BDE-22	BDE-13	BDE-8	BDE-5	BDE-3	BDE-2	BDE-1
*E*_HOMO_	−0.24561	−0.24321	−0.24051	−0.23786	−0.2419	−0.23279	−0.23812	−0.23537
*E*_LUMO_	−0.05789	−0.05047	−0.04592	−0.0443	−0.04342	−0.03813	−0.03744	−0.03597

## Supplementary Materials

Supplementary materials can be found at http://www.mdpi.com/1422-0067/17/6/927/s1.

## Abbreviations

PBDEs	Polybrominated diphenyl ethers
RoHs	The Restriction of the use of Certain Hazardous Substances in Electrical and Electronic Equipment
PBB	Polybrominated biphenyls
DFT	Density functional theory
PM3	Parametrized model 3
AM1	Austin model 1
MNDO	Modified neglect of differential overlap
TCDD	Tetrachlorodibenzodioxin
PCDD	Polychlorinated dibenzo-p-dioxins
PCDFs	Polychlorinated dibenzofurans
B3LYP	Becke-Lee-Yang-Parr hybrid method
CPCM	Conductor-like polarizable continuum model
PES	potential energy surface scan
TS	transition state
IRC	intrinsic reaction coordinated
EA	Electron affinities
EA_Ada_	Adiabatic electron affinities
EA_ver_	Vertical electron affinities
ZPC	Zero-point corrections
LUMO	The lowest unoccupied molecular orbital
HOMO	The highest occupied molecular orbital
RMSE	Root mean square error
