# Supplementary material for: Theoretical Studies on Structures, Properties and Dominant Debromination Pathways for Selected Polybrominated Diphenyl Ethers
Source: Int J Mol Sci. 2016 Jun 16;17(6):927. doi: 10.3390/ijms17060927 (PMC4926460; doi:10.3390/ijms17060927)
Supplement: Supplementary file 1 [file ijms-17-00927-s001.pdf]

Supplementary Materials: Theoretical Studies on Structures, Properties and Dominant Debromination Pathways for Selected Polybrominated Diphenyl Ethers

Lingyun Li, Jiwei Hu, Xuedan Shi, Wenqian Ruan, Jin Luo and Xionghui Wei

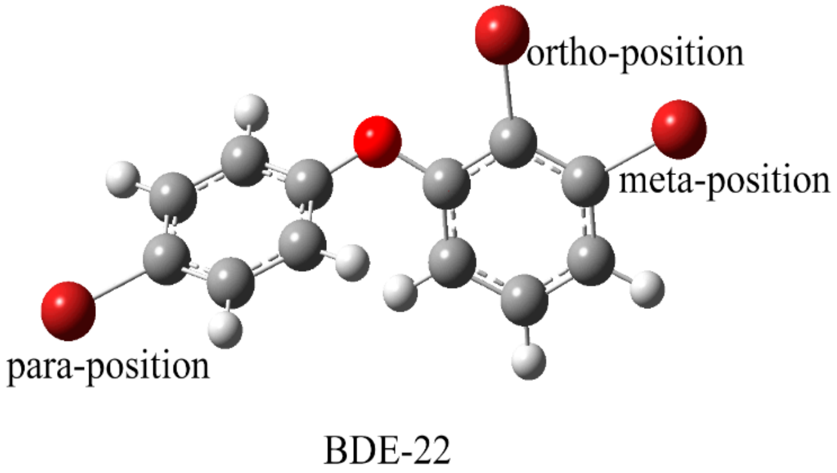

(**a**)

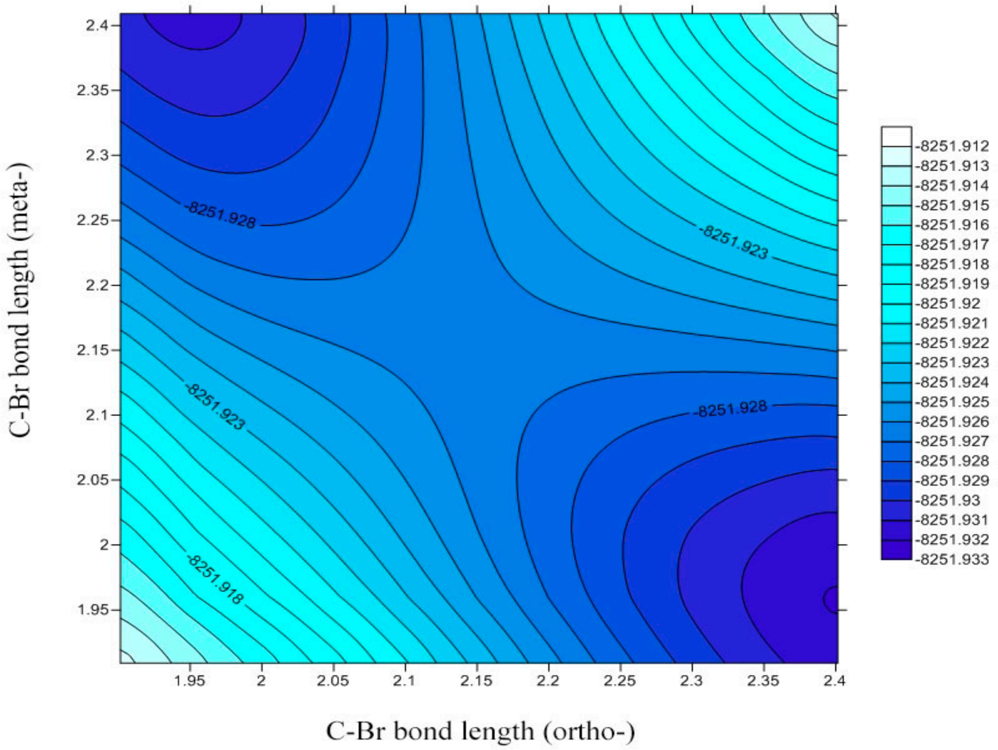

(**b**)

Figure S1. *Cont.*

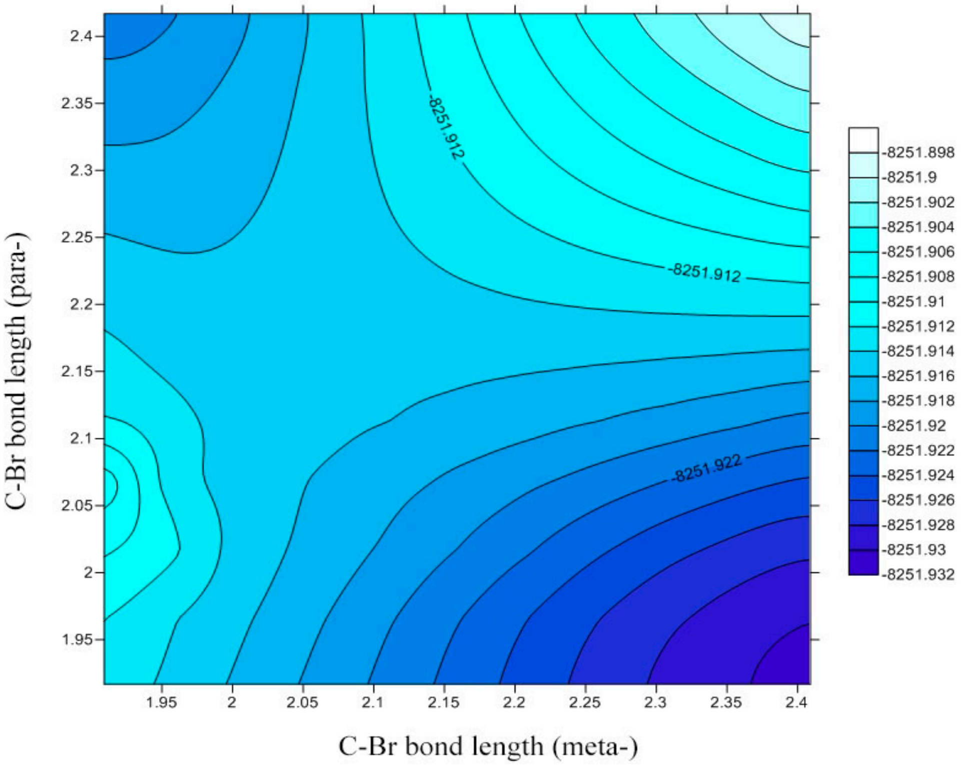

(**c**)

Figure S1. Potential energy surface (in hartree) for 2,3,4′-tribromodiphenyl ether as function of the two C–Br bond lengths ((**a**) molecular structure of BDE-22 and definition of C-Br bond; (**b**) bonds at the ortho- and the meta- position; (**c**) bonds at the meta- and the para- position).

The grid file of the potential energy surface was created by the Kriging method with linear variogram model (slope = 1, anisotropy ratio = 1, angle = 0) and the contour XY grid map was drawn using Surfer 10 software (Golden software, Inc., Golden, CO, USA).

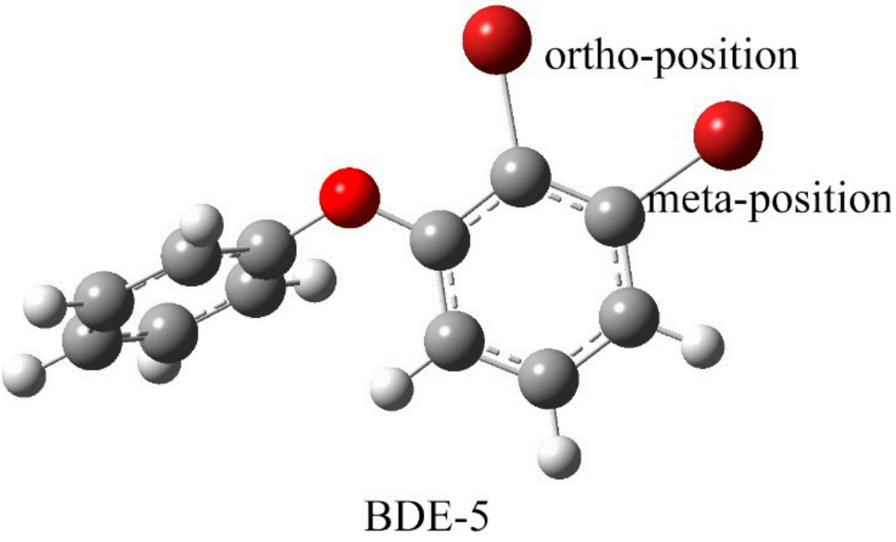

(**a**)

Figure S2. *Cont.*

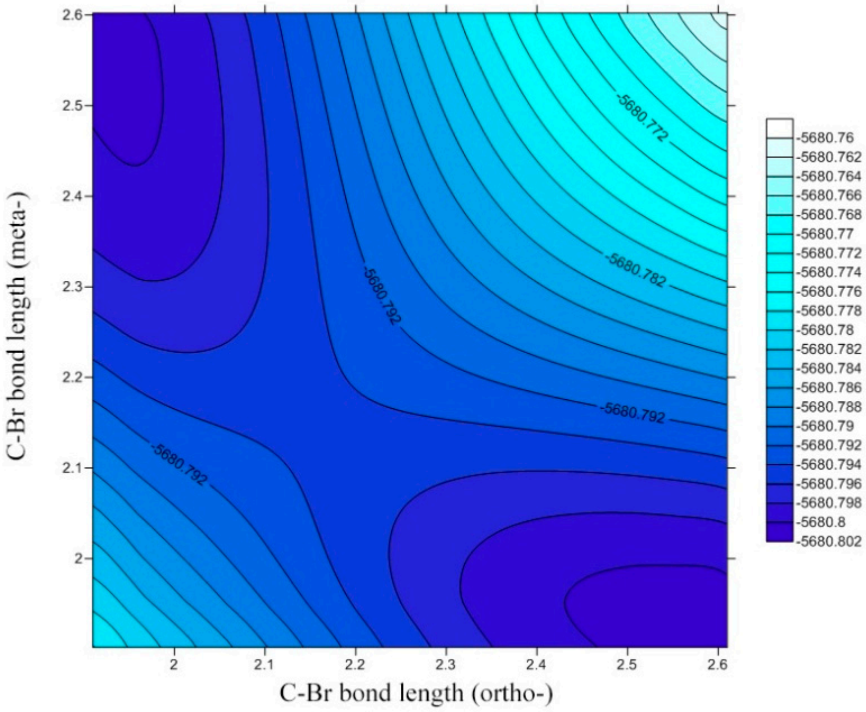

(**b**)

Figure S2 Potential energy surface (in hartree) for 2,3-dibromodiphenyl ether as function of the two C–Br bond lengths ((**a**) molecular structure of BDE-5 and definition of C-Br bond; (**b**) bonds at the ortho- and the meta- position).

The grid file of the potential energy surface was created by the Kriging method with linear variogram model (slope = 1, anisotropy ratio = 1, angle = 0) and the contour XY grid map was drawn using Surfer 10 software (Golden software, Inc.).

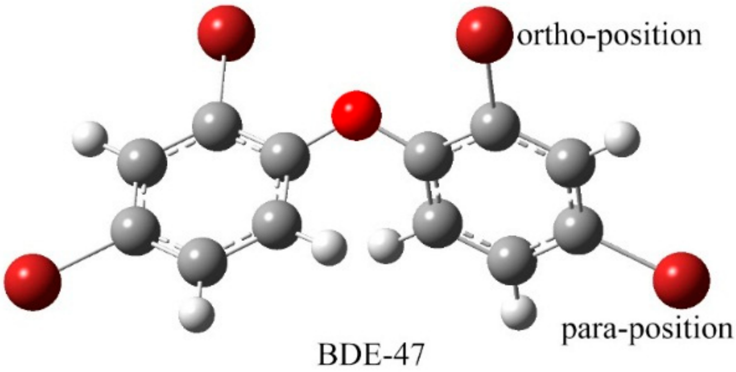

(**a**)

Figure S3. *Cont.*

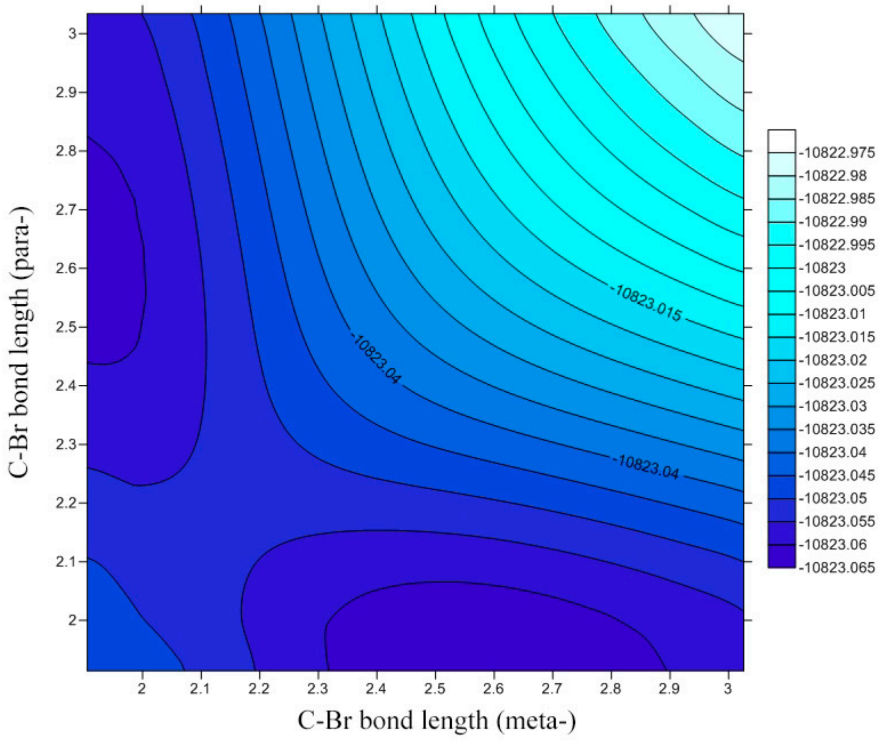

(**b**)

Figure S3. Potential energy surface (in hartree) for 2,2′, 4,4′-tetrabromodiphenyl ether as function of the two C–Br bond lengths ((**a**) molecular structure of BDE-47 and definition of C-Br bond; (**b**) bonds at the meta- and the para- position).

The grid file of the potential energy surface was created by the Kriging method with linear variogram model (slope = 1, anisotropy ratio = 1, angle = 0) and the contour XY grid map was drawn using Surfer 10 software (Golden software, Inc.).

Table S1. Thermodynamic data (hartrees) of BDE-22 calculated with B3LYP/6-311+G(d) in gas-phase. (Temperature of 298.150 Kelvin. Pressure of 1.00000 Atm.)

Congener	*E*	*H*	*G*
BDE-22	−8259.103692	−8259.088181	−8259.150652
anionic state I	−8259.145470	−8259.129091	−8259.194947
anionic state II	−8259.142845	−8259.126380	−8259.193342
anionic state III	−8259.132438	−8259.115923	−8259.183070
TS1	−8259.137981	−8259.122139	−8259.187250
TS2	−8259.121751	−8259.105519	−8259.172027

Table S2. Thermodynamic data (hartrees) of BDE-5 calculated with B3LYP/6-311+G(d) in gas-phase. (Temperature of 298.150 Kelvin. Pressure of 1.00000 Atm.)

Congener	*E*	*H*	*G*
BDE-5	−5685.550450	−5685.536424	−5685.594495
anionic state I	−5685.586582	−5685.571724	−5685.632847
anionic state II	−5685.585402	−5685.570488	−5685.632149
TS1	−5685.579694	−5685.565402	−5685.625471

Table S3. Thermodynamic data (hartree) of BDE-47 calculated with B3LYP/6-311+G(d) in gas-phase. (Temperature of 298.150 Kelvin. Pressure of 1.00000 Atm.)

Congener	*E*	*H*	*G*
BDE-47	−10,832.657436	−10,832.640342	−10,832.707715
anionic state I	−10,832.701399	−8259.129091	−8259.194947
anionic state II	−10,832.695098	−10,832.677021	−10,832.749899
TS1	−10,832.687913	−10,832.670306	−10,832.740132

Molecular Geometries

BDE-22 neutral

C	−3.77087900	−0.60800800	1.15290600
C	−4.17110200	−0.02646900	−0.04628200
C	−3.23304500	0.40207900	−0.98196700
C	−1.87518200	0.24940800	−0.71337100
C	−1.47134800	−0.32144000	0.49253400
C	−2.41200100	−0.75323900	1.42293300
O	−0.14062200	−0.56940200	0.77922100
C	0.81155900	0.39573100	0.57603200
C	0.55790300	1.74832700	0.79778900
C	1.57940900	2.67791200	0.64141700
C	2.85716600	2.27083400	0.27425700
C	3.11343300	0.91701400	0.06162700
C	2.09735000	−0.03367300	0.20591800
Br	2.38851800	−1.89168700	−0.07378400
Br	4.88827300	0.42082800	−0.43666300
Br	−6.03973900	0.18520000	−0.41718000
H	−4.50666300	−0.94313900	1.87363000
H	−3.55390000	0.84171500	−1.91854800
H	−1.13765600	0.56709200	−1.44148500
H	−2.07464200	−1.20607300	2.34815500
H	−0.43521600	2.06114700	1.09697300
H	1.38101800	3.72963100	0.81660400
H	3.65563100	2.99203400	0.15507700

BDE-22 anion (elongated at ortho- brominated position)

C	−3.42937700	0.57141900	1.12760600
C	−3.56139500	0.18471900	−0.20257600
C	−2.48520800	0.25904900	−1.07727700
C	−1.25686200	0.73665200	−0.62438100
C	−1.11177600	1.13803600	0.70606800
C	−2.20251600	1.04420600	1.57729500
O	0.01829500	1.68754600	1.22881300
C	1.23600000	1.64939300	0.53816400
C	1.85055300	2.88495100	0.33487600
C	3.09724000	2.92974300	−0.29079800
C	3.70256400	1.75162400	−0.72615600
C	3.04108300	0.54349200	−0.49417800
C	1.83088500	0.45914500	0.14373600
Br	1.19797800	−1.60986500	1.55530300
Br	3.93149200	−1.07413600	−1.17187400
Br	−5.26332000	−0.47056100	−0.83793200
H	−4.26785200	0.49659200	1.81021600
H	−2.59074700	−0.05886600	−2.10789700
H	−0.41206600	0.77341000	−1.29878000
H	−2.06678800	1.33594400	2.61249000
H	1.35314200	3.79178900	0.66692000
H	3.59344100	3.88310600	−0.44796900
H	4.66211800	1.77273400	−1.23217000

BDE-22 anion (elongated at meta- brominated position)

C	−3.77171400	0.26422800	1.19049600
C	−3.88618500	−0.02124000	−0.16751300
C	−2.79302200	0.10115300	−1.01569500
C	−1.56513900	0.52274400	−0.50727400
C	−1.43913700	0.81795500	0.85245000
C	−2.54694300	0.68241600	1.69677100
O	−0.28654000	1.24710300	1.44055800
C	0.85427300	1.45785800	0.65710800
C	1.06342400	2.73285000	0.13755400
C	2.21898800	2.99602000	−0.59984900
C	3.15639100	1.98184000	−0.81073300
C	2.93853800	0.71818700	−0.27805200
C	1.81013000	0.45705500	0.44839500
Br	1.48836200	−1.31024600	1.22793500
Br	4.95152800	−0.87821600	−0.93509300
Br	−5.58437400	−0.59590300	−0.87700800
H	−4.62612500	0.16097700	1.84911000
H	−2.88503800	−0.13364800	−2.06949300
H	−0.70863500	0.60976800	−1.16385900
H	−2.42949700	0.90520200	2.75142400
H	0.31926400	3.50127600	0.32138200
H	2.38290400	3.99297800	−1.00384100
H	4.05876300	2.17299800	−1.38534300

BDE-22 anion (elongated at para- brominated position)

C	3.42010600	−0.54202700	−1.20516600
C	4.10386100	−0.47294700	−0.00001100
C	3.42034800	−0.54216000	1.20526800
C	2.02613400	−0.68143700	1.21447600
C	1.35100000	−0.74118700	0.00025000
C	2.02590100	−0.68131500	−1.21411000
O	−0.05190100	−0.91771900	0.00037100
C	−0.85482000	0.17015800	0.00028800
C	−0.37834200	1.48523100	0.00041800
C	−1.27016900	2.55057000	0.00035300
C	−2.64442000	2.33697700	0.00016100
C	−3.12265200	1.02776000	0.00003800
C	−2.24791300	−0.05930500	0.00010200
Br	−2.85950600	−1.86489000	−0.00005400
Br	−5.02674700	0.79075600	−0.00022300
Br	6.68980300	0.45442600	−0.00020800
H	3.96102300	−0.48513500	−2.14692400
H	3.96145000	−0.48540200	2.14692700
H	1.46537700	−0.74329100	2.14403500
H	1.46495200	−0.74309500	−2.14355800
H	0.69062000	1.65472400	0.00056100
H	−0.88649600	3.56559500	0.00045100
H	−3.34020400	3.16621600	0.00010500

TS1 of anionic state I to II for BDE-22

C	−3.43636600	0.64208900	1.12353200
C	−3.54634700	0.32453600	−0.22739000
C	−2.43671200	0.36474100	−1.06160700
C	−1.19596700	0.73686300	−0.54660900
C	−1.07281000	1.06631500	0.80573600
C	−2.19828200	1.01030200	1.63586200
O	0.08378200	1.47601700	1.39499000
C	1.27376900	1.52417400	0.65274500
C	1.71260400	2.78387500	0.24394900
C	2.91975000	2.90145200	−0.45027700
C	3.66611700	1.75990000	−0.75278400
C	3.18394400	0.53554900	−0.31277200
C	2.04302900	0.40281600	0.37291100
Br	1.42985700	−1.48769200	1.31770700
Br	4.41769300	−1.21151400	−1.05927100
Br	−5.26399600	−0.18142000	−0.94763600
H	−4.30329400	0.59906600	1.77251300
H	−2.52566700	0.10180400	−2.10904100
H	−0.32673900	0.75180400	−1.19139800
H	−2.08263000	1.25218300	2.68643800
H	1.10496600	3.65535900	0.46968300
H	3.27313900	3.88354900	−0.75633000
H	4.59526600	1.82988800	−1.31248900

TS2 of anionic state II to III for BDE-22

C	3.41953500	−0.15770200	−1.20732700
C	4.13377800	0.00027200	−0.02558200
C	3.50523300	−0.11674900	1.20791000
C	2.12710900	−0.34013000	1.26254300
C	1.40180300	−0.46824800	0.07868700
C	2.04231200	−0.38237400	−1.15752100
O	0.04055100	−0.75042700	0.13490700
C	−0.87285000	0.27792700	0.05708600
C	−0.49831300	1.62209900	−0.00201400
C	−1.47600400	2.61328100	−0.07622800
C	−2.82637700	2.27884800	−0.09246000
C	−3.17503500	0.93373800	−0.02955500
C	−2.23279800	−0.05637000	0.04496700
Br	−2.72166700	−1.99297300	0.14869300
Br	−5.18978800	0.53507700	−0.06070500
Br	6.31343300	−0.14818800	−0.10281900
H	3.92374400	−0.09640900	−2.16786800
H	4.07540800	−0.02289200	2.12814200
H	1.60785800	−0.41057600	2.21413600
H	1.45856000	−0.48495500	−2.06790900
H	0.55304100	1.88417500	0.00951000
H	−1.17523800	3.65695700	−0.12154700
H	−3.59405500	3.04394000	−0.15317500

Figure S4. The Cartesian coordinates of optimized BDE-22 geometries (calculated with B3LYP/6-311+G(d) in gas-phase).

BDE-5 neutral

C	−5.13229100	−0.24997000	−0.80560500
C	−5.51386600	0.24617000	0.43969400
C	−4.54212100	0.51601400	1.40387400
C	−3.19457400	0.29383900	1.13004100
C	−2.82833800	−0.19294000	−0.12347800
C	−3.78589700	−0.46987600	−1.09375900
O	−1.50824000	−0.51055200	−0.41467600
C	−0.52477700	0.43434000	−0.31428500
C	−0.77257500	1.80038600	−0.44461600
C	0.28259200	2.70401800	−0.38584200
C	1.58774600	2.26158800	−0.20527300
C	1.83696400	0.89509900	−0.08191500
C	0.79049600	−0.03025300	−0.13199800
Br	3.64993500	0.34662100	0.16459300
Br	1.07343200	−1.90393000	0.03357500
H	−5.88227800	−0.46786100	−1.55882700
H	−6.56205500	0.41634800	0.66106900
H	−4.83309700	0.89086700	2.37974900
H	−2.43467400	0.48664100	1.87920300
H	−3.46892400	−0.86032300	−2.05418800
H	−1.78795600	2.14532000	−0.59566800
H	0.08593100	3.76561100	−0.48952900
H	2.41097400	2.96312800	−0.16131300

BDE-5 anion (elongated at ortho- brominated position)

C	−4.70861500	−0.23386400	0.14576400
C	−4.52361800	−0.19742600	1.52951000
C	−3.30141400	0.24138000	2.03518100
C	−2.27353100	0.64677000	1.18536200
C	−2.47008000	0.61106500	−0.19757200
C	−3.69202300	0.16481800	−0.71365100
O	−1.56051600	1.04473800	−1.11808700
C	−0.24542500	1.34700800	−0.75159200
C	0.16764800	2.65501100	−1.00636200
C	1.48490400	3.02211600	−0.72687200
C	2.36603700	2.09254600	−0.17645200
C	1.89887100	0.79682400	0.05577100
C	0.62182900	0.39376700	−0.23359200
Br	3.18410100	−0.44874500	0.87657700
Br	0.10448700	−2.05458200	−0.89039900
H	−5.64894300	−0.58649200	−0.26925200
H	−5.31482900	−0.51800900	2.20049400
H	−3.13435500	0.26258400	3.10853900
H	−1.32111200	0.96424000	1.58870100
H	−3.81416100	0.12565500	−1.79046700
H	−0.54066500	3.36767500	−1.41945100
H	1.82302100	4.03452100	−0.92892400
H	3.38708200	2.36827700	0.06584500

BDE-5 anion (elongated at meta- brominated position)

C	−5.06292600	−0.51060400	0.09084400
C	−4.90479700	−0.37295200	1.47171600
C	−3.68326000	0.07397800	1.97154900
C	−2.62711200	0.38778900	1.11680900
C	−2.79738800	0.24919100	−0.26289800
C	−4.01907800	−0.20322600	−0.77411400
O	−1.82938700	0.53147700	−1.18708500
C	−0.60813100	1.06673300	−0.77067900
C	−0.48663100	2.45288200	−0.70365000
C	0.73749200	3.02398300	−0.35222400
C	1.83285800	2.20459500	−0.06763900
C	1.70021900	0.82504600	−0.14755700
C	0.50428400	0.26131800	−0.49801600
Br	3.98036200	−0.31879700	0.63585300
Br	0.30314600	−1.67896100	−0.64918500
H	−6.00615600	−0.86306900	−0.31699400
H	−5.71959000	−0.61663300	2.14626100
H	−3.53952200	0.17652500	3.04318300
H	−1.67744200	0.72405900	1.51403000
H	−4.12391200	−0.31122900	−1.84827900
H	−1.35421200	3.06437300	−0.93066400
H	0.83047400	4.10715600	−0.30330600
H	2.78949100	2.63610600	0.21421400

TS1 of anionic state I to II for BDE-5

C	−5.02145500	−0.55085200	0.10550600
C	−4.84664100	−0.46353900	1.48851500
C	−3.63043600	−0.00121500	1.98742400
C	−2.59759700	0.37776200	1.13105300
C	−2.78419800	0.29095800	−0.25102500
C	−4.00057600	−0.17829700	−0.76083100
O	−1.85159000	0.66067800	−1.17693800
C	−0.60472400	1.14619100	−0.76342400
C	−0.42417600	2.53028500	−0.78315300
C	0.81716100	3.07100400	−0.43825600
C	1.86437500	2.22915500	−0.05521600
C	1.62818000	0.86199500	−0.06205500
C	0.45474300	0.32201600	−0.40757100
Br	3.36917100	−0.32777100	0.74384500
Br	0.16673600	−1.85702100	−0.68191500
H	−5.95900200	−0.91798600	−0.30299900
H	−5.64259800	−0.76025300	2.16458300
H	−3.47168500	0.06009300	3.06028900
H	−1.65003800	0.71809700	1.52880600
H	−4.11736100	−0.25052400	−1.83672900
H	−1.25725100	3.16781200	−1.06515500
H	0.96402800	4.14844200	−0.46739200
H	2.83143600	2.63201600	0.23488500

Figure S5. The Cartesian coordinates of optimized BDE-5 geometries (calculated with B3LYP/6-311+G(d) in gas-phase).

BDE-47 neutral

C	−2.30981000	0.72112800	−0.56595700
C	−3.55230100	0.10829500	−0.41907500
C	−3.66167200	−1.02022600	0.38584400
C	−2.55360000	−1.53779200	1.05039800
C	−1.32016000	−0.91291100	0.90391200
C	−1.18511400	0.21383200	0.09199600
O	0.00057000	0.89803900	−0.02158400
C	1.19112200	0.21980400	−0.12247000
C	1.34377200	−0.89064300	−0.95344400
C	2.58125900	−1.50940700	−1.09013900
C	3.67583400	−1.00142900	−0.39609500
C	3.54900300	0.11092700	0.42842900
C	2.30204300	0.71744400	0.56511300
Br	2.12587900	2.23990800	1.69778300
Br	5.38632000	−1.84128500	−0.57674200
Br	−2.15804000	2.26545900	−1.67236200
Br	−5.36640100	−1.86895100	0.57977700
H	−4.41593900	0.50954100	−0.93249400
H	−2.64879700	−2.41037300	1.68449200
H	−0.45279700	−1.29665700	1.42877200
H	0.48638400	−1.26574100	−1.50061800
H	2.69050700	−2.36928100	−1.73913000
H	4.40228900	0.50446200	0.96460700

BDE-47 anion (elongated at para- brominated position)

C	2.45617700	−0.75663600	−0.56745400
C	3.55459100	0.06598200	−0.34509100
C	3.34199800	1.38730500	0.03284200
C	2.05558300	1.88816400	0.18715200
C	0.96467100	1.05453500	−0.03884800
C	1.14471800	−0.27991400	−0.41719700
O	0.12775400	−1.14680100	−0.64731000
C	−1.19301700	−0.68725100	−0.57848300
C	−1.79285400	−0.18713700	−1.73315500
C	−3.12361500	0.23304700	−1.69218200
C	−3.84132800	0.14061700	−0.50818700
C	−3.26260300	−0.36457500	0.64523800
C	−1.93078200	−0.78191200	0.60201700
Br	−1.08902600	−1.50069800	2.19700200
Br	−6.21064200	1.39402900	−0.43969700
Br	2.75373800	−2.57096700	−1.09282000
Br	4.85881300	2.52951400	0.34186600
H	4.55620200	−0.32427800	−0.46762600
H	1.89418100	2.91719500	0.48409800
H	−0.04168800	1.43655900	0.08114300
H	−1.20304700	−0.13768700	−2.64591500
H	−3.59455700	0.63230300	−2.58687300
H	−3.82526200	−0.43874400	1.57147900

BDE-47 anion (elongated at ortho- brominated position)

C	−2.26632000	1.21753700	0.20168900
C	−3.46771000	0.62512000	−0.17062200
C	−3.43753000	−0.64539100	−0.73226300
C	−2.23733300	−1.32081900	−0.91145300
C	−1.04347700	−0.71667500	−0.53159200
C	−1.03391500	0.56755900	0.02342100
O	0.07491700	1.24436300	0.40428400
C	1.37653300	0.84391700	0.05764300
C	2.11705800	1.80870200	−0.63104600
C	3.44902900	1.55493400	−0.95059600
C	4.00019200	0.33335800	−0.57336700
C	3.26587600	−0.62927200	0.11412300
C	1.94194500	−0.35941300	0.43444000
Br	1.00179400	−2.35801800	1.97181400
Br	5.85978200	−0.02005900	−1.02712600
Br	−2.31574800	2.97674300	0.96355900
Br	−5.09597500	−1.47493000	−1.25906500
H	−4.40130800	1.15190200	−0.02537100
H	−2.22120400	−2.32346100	−1.32049500
H	−0.11459700	−1.26294900	−0.61365500
H	1.65125200	2.75129700	−0.90565600
H	4.03966100	2.29202600	−1.48108700
H	3.71591200	−1.57184900	0.40833200

TS1 of anionic state I to II for BDE-47

C	−2.39706200	0.83719600	−0.55472200
C	−3.53595200	0.05464000	−0.40226700
C	−3.38990800	−1.29298400	−0.09424900
C	−2.13065400	−1.85823400	0.06174900
C	−0.99985000	−1.06326700	−0.09251000
C	−1.11047800	0.29659400	−0.40298200
O	−0.05557900	1.12497400	−0.57601900
C	1.25037500	0.61144300	−0.52844700
C	1.87235600	0.31642100	−1.74174400
C	3.18713000	−0.14740700	−1.71994900
C	3.82484400	−0.29502300	−0.49881400
C	3.24045100	−0.00136800	0.71874900
C	1.93594700	0.46001500	0.66908600
Br	0.92520300	1.01262000	2.53294300
Br	5.92883400	−1.14600000	−0.58141500
Br	−2.60427700	2.68790700	−0.99164700
Br	−4.96339400	−2.38306700	0.11501100
H	−4.51603500	0.49610400	−0.52461800
H	−2.02207600	−2.90708700	0.30967600
H	−0.01536100	−1.49265400	0.04287100
H	1.32781500	0.45621300	−2.67536900
H	3.70722000	−0.38478200	−2.64303500
H	3.77060100	−0.11824900	1.66241700

Figure S6. The Cartesian coordinates of optimized BDE-47geometries (calculated with B3LYP/6-311+G(d) in gas-phase).
